# In-depth investigation the size effect of zinc oxide nanostructures on the photodegradation of different dyes under UV-irradiation: anticancer application

**DOI:** 10.1038/s41598-025-16270-4

**Published:** 2025-08-28

**Authors:** Moustafa. E. Elsisi, Mai Mohamed Mostafa, Hanan Abdella, Aia E. Khalil, Abdelfatah Salah Soror

**Affiliations:** 1 Physics Department, Faculty of Science, Zagazig University, Zagazig, 44519 Egypt; 2 Botany and Microbiology Department, Faculty of Science, Zagazig University, Zagazig, 44519 Egypt; 3 Department of Environmental Biotechnology, Genetic Engineering and Biotechnology Research Institute, Sadat City University, Sadat City, 32897 Egypt

**Keywords:** Organic dyes, ZnQ QD_s_, Green synthesis, Photocatalytic activity, Anticancer activities, Cancer, Cell biology, Materials for energy and catalysis

## Abstract

The aim of this research was to prepare a different particle sizes of zinc oxide nanostructures by two different methods. The zinc oxide nanoparticle (ZnO NP_s_) was successfully prepared by a green synthesis technique but the zinc oxide quantum dot (ZnO QD_s_) was successfully prepared by a chemical method. The structure, composition and morphology of the prepared different shapes of ZnO nanostructures have been characterized by the means of X-ray diffractograms (XRD), high resolution transmission electron microscope (HRTEM), Energy Dispersive x-ray (EDX), UV-Vis spectroscopy and Fourier transform infrared spectroscopy (FTIR). From UV-Vis spectroscopy studies we noticed that the optical band gap energy of ZnO nanostructures was decreased by increasing an irradiation time. The removal of complex organic contaminants and pollutants from water, the heterogeneous photocatalytic degradation of methylene blue (MB), Fluorescein and Rhodamine 6G (Rh 6G) dyes were studied using ZnO NP_s_ and ZnO QD_s_ as a derived catalyst. We had studied the impact of ZnO NP_s_ and ZnO QD_s_ as a catalyst to enhance the photocatalytic activity of different organic dyes under UV-Vis irradiation and we observed that the photodegradation percentage of organic dyes was rapidly increased by increasing UV irradiation time in both two shapes of ZnO nanostructures. ZnO QD_s_ behave as the best photocatalyst for successfully photodegraded due to the smallest size of ZnO QD_s_ has a higher photocatalytic activity than the large particle size of ZnO NP_s_. So, it is better to use the ZnO QD_s_ as a removal dyes and pollutants in the wastewater application. Also, we have assessed the cytotoxicity of ZnO NP_s_ and ZnO QD_s_ against two cell lines, (T-47) breast cancer carcinoma, and (DU-145) prostate cancer cell compared to Human skin fibroblast (HSF). The proliferation of cancer cells using MTT assay clarified that both cancer cells (T-47), (DU-145) as well as (HSF) normal cell line are regularly inhibited as they grow on different concentrations of ZnQ QD_s_ and ZnQ NP_s_. The maximum inhibitory effect of both were recorded at concentration of 100 µg/ml (62.63, 79.72 and 42.59% and 72.68, 83.28, 18.12 µg/ml) in case of ZnQ QD_s_ and ZnQ NP_s_ respectively. It was cleared that ZnQ NP_s_ was more potent for test cancer cell lines, this was confirmed by IC_50,_ since it was (18.12,13.3,74.86) in ZnO NP_s_ compared with (42.59,17.05 and 76.4) in ZnQ QD_s_ respectively. Finally, it was proved that the ZnO NP_s_ behave as a good anticancer nanomaterial than ZnO QD_s_. This means ZnO NP_s_ are superior for anticancer applications if compared with ZnO QDs.

## Introduction

Numerous different technologies have been applied to remove organic dyes from wastewater such as adsorption, coprecipitation, advanced oxidation process (AOP), ozonation, membrane filtration and biological methods. Many technologies, including adsorption, membrane filtration, ozonation, coprecipitation, advanced oxidation process (AOP), and biological approaches, have been used to remove organic dyes from wastewater. AOP is noticeable because it could quickly remove various types of dyes. Among AOP technique catalyst is gaining attention as it can remove not only organic dyes but also other organic pollutants, the ability of AOP to rapidly eliminate different kinds of dyes makes it distinctive. AOP catalysts are becoming more popular because they can eliminate various organic contaminants in addition to organic dyes^1,2^. Zinc oxide nanopowders (ZnO-NP_s_) are an important metal oxide due to its interesting properties and wide applications in various fields, nanosized ZnO particles are an important inorganic semiconductor material with a hexagonal wurtzite crystal structure which has a wide and direct band gap (nearly 3.37 eV) and large excitonic binding energy (60meV) at ambient temperature^46^. The various methods used for synthesis of these ZnO-NP_s_ include solvothermal and hydrothermal synthesis, the solgel method has gained much interest among researchers as it offers controlled consolidation, shape modulation, patterning of the nanostructures and low processing temperature^47^. Water pollution causes great damage to ecosystems, human health, as well as the sustainable economic and social development because the pollutant complex cause difficulty in decontamination by conventional water treatment processes. Water pollution seriously harms ecosystems, human health, and sustainable economic and social development. Because the pollutant complex makes it impossible for traditional water treatment methods to decontaminate these complexes. Hence, developing an effective and facile way to degrade pollutants has become an active area in environmental research. Recently, inorganic nanomaterials have attracted numerous attentions because of their controllable shapes and sizes, as well as their effective photocatalytic activities, such as those in metal oxide semiconductors (e.g. TiO_2_, ZnO) or narrow band gap semiconductors (e.g. Ag_3_PO_4_)^3^. Removal of toxic organic dyes from water have been provided the challenge for continued fundamental and applied research in the field of photocatalysis, synthesis of a new catalyst composite with unique nano-structure and photoelectronic properties has been received considerable interest in the recent years^4,5^. The dyes discharge in water is the foremost cause of dangerous environmental risks. Moreover, discarding toxic dyes into commonly used water allows for hazardous effect to the human and the aquatic medium. Industrial effluents and wastewater generated from organic dye usage are typically toxic to aquatic organisms and humans, therefore the efficient and environmentally friendly removal of dyes from wastewater is of utmost important for clean production practices^48^. Conventional treatment methods for organic dye wastewater are expensive, time-consuming, prone to secondary pollution, and often result in incomplete degradation^48^. The use of ZnO nanoparticles in photocatalysis and their potential cytotoxicity towards cancer cells show the diverse range of applications that nanomaterials can have in environmental improvement and health care. The challenges faced by ZnO nanoparticles, such as the fast recombination of electron -hole pairs and poor absorption of visible light, highlight the importance of exploring nanocomposites and modifications to enhance their performance^49,51^. A wide spectrum of semiconductors has been used as catalysts to achieve considerable mineralization of dyes from wastewater^6^. Photocatalysis has deserved a growing interest as an important solution to clean water, air and soils from toxic contaminants over the last decades^7,8^. One of key materials in this connection became titanium dioxide (TiO_2_)^9^, which has shown high effectiveness in the environmental process. Its main shortcoming, however, consists in a relatively rapid charge recombination reducing the energetic efficiency and activation under UVA light illumination. Much effort has been done in the solution of the above problem by doping titanium dioxide with metal cation (Ag, Fe, V, AU, Pt, etc.) and non-metallic element (N, S, C, B, P, etc.). They have observed a moderate decrease of the rhodamine 6G (Rh 6G), Fluorescein and Methylene blue (MB) decomposition rate under UV light illumination at (533 nm), (499 nm) and (664 nm)^10^.

Cancer is a complex group of illnesses defined by the uncontrolled proliferation of aberrant cells, which may give rise to the formation of tumors capable of infiltrating adjacent organs and inducing significant detrimental consequences in affected individuals, possibly resulting in mortality^11^. there has been a growing popularity in the use of nanotechnology due to its potential applications in cancer research. It has been shown that encapsulating chemotherapeutic medications has proven to be an effective method for targeted delivery to the tumor location. According to^12^, there is evidence to suggest that the occurrence of adverse effects may be reduced or eliminated. In addition, the use of nano materials (NMs) enables a higher degree of permeability to the tumor site, in comparison to. The provision of pharmaceutical substances without charge^13,14^. The generation of a substantial quantity of reactive oxygen species (ROS) in cancerous cells in comparison to healthy cells leads to an increased ROS concentration, subsequently resulting in impaired mitochondrial function and triggering the intrinsic mitochondrial apoptotic pathway^15^. Nanoparticles (NPs) possess the capability of functionalization, which allows them to target malignant cells selectively. The increased cellular penetration and DNA binding of the complex might perhaps be ascribed to the robust Zn-O bond^16^. There was a dose-dependent increase in vitro cytotoxicity against many cancer cell lines when phytofabricated ZnO NPs were used in anticancer investigations. Zinc oxide nanoparticles have garnered considerable attention in several sectors, including optics, electrics, packaged foods, and medicine, owing to their favorable attributes such as biocompatibility, low cytotoxicity, and cost effectiveness. Acritical element of ZnO nanoparticles pertains to their capacity to induce cellular death via the facilitation of reactive oxygen species (ROS) production and the liberation of zinc ions (Zn^+2^), which exhibit cytotoxic qualities^50^.

The purpose of this work was to improve the total photodegradation efficiency and photocatalytic activity of several organic dyes (MB, fluorescein, and Rh 6G) by producing ZnO nanoparticles and ZnO quantum dots using two distinct methods. The production of a novel ZnO nanostructure has been explained. The morphologies, structures, optical characteristics, and photocatalytic characteristics are all carefully analyzed. Where, the XRD confirms the formation of ZnO nanoparticle in the range of crystalline size (35 nm) and the crystalline size range of ZnO quantum dot is 7.5 nm. ZnO nanoparticle was used in this study to enhance the photocatalytic degradation of Fluorescein and Rh 6G dyes and ZnO quantum dot was used to enhance the photocatalytic activity of MB and Rh 6G dyes under UV-irradiation. In the present work, the new in this paper is that we used the Fluorescein, and Rh 6G dyes with ZnO nanostructures to improve the photocatalytic activity of these dyes to improve the higher photodegradation performance and lower catalyst dosage requirements. From the photocatalytic data, we observed that the ZnO QD_s_ has the powerful effect to increase the photodegradation rate of MB and Rh 6G dyes than ZnO NP_s_. This is due to the small particle size of ZnO QD_s_ to be active as a removal dye. Also, we had applied the prepared ZnO nanostructures in an anticancer application.

## Materials and methods

### Algal collection

*Padina pavonica* was collected from the coast of Abu-Qir at Alexandria Province – Egypt. The selected alga in the present investigation is a member of brown algae, Division Phaeophyta. The collected alga sample was stored in plastic bags with water to prevent evaporation, and transported to the laboratory under iced conditions. The alga species was identified based on the schemes reported in the literature^17,18^. The selected alga was washed with sea water to remove unwanted impurities and other debris. Then washed with tap water for 3 to 4 times to remove epiphytes, sand particles, and other fauna and then washed thoroughly with fresh water to remove salts, and stored at 20 °C until compound extraction. They were shade and air dried. Then cut into small pieces and kept in hot air oven for one day at 40˚C, and it was made into coarse powder with the help of mixer grinder. Grind dried seaweed using ceramic mortar into a fine powder and stored in air tight bottles till the following analysis were performed^19^.

### Preparation of aqueous algal extract

It was carried out according to^19^. Five grams of dried finely powdered algal tissue was taken in a flask. Add 200 ml of distilled water and left for 12 h. The mixture was heated on a mantle with continuous stirring at 30–40 ˚C for 20 min. The water extract was filtered through filter paper and was centrifuged for 15 min. The water extract was kept in refrigerator when not in use.

### Green synthesis of ZnO nanoparticles

**Fig. 1:**
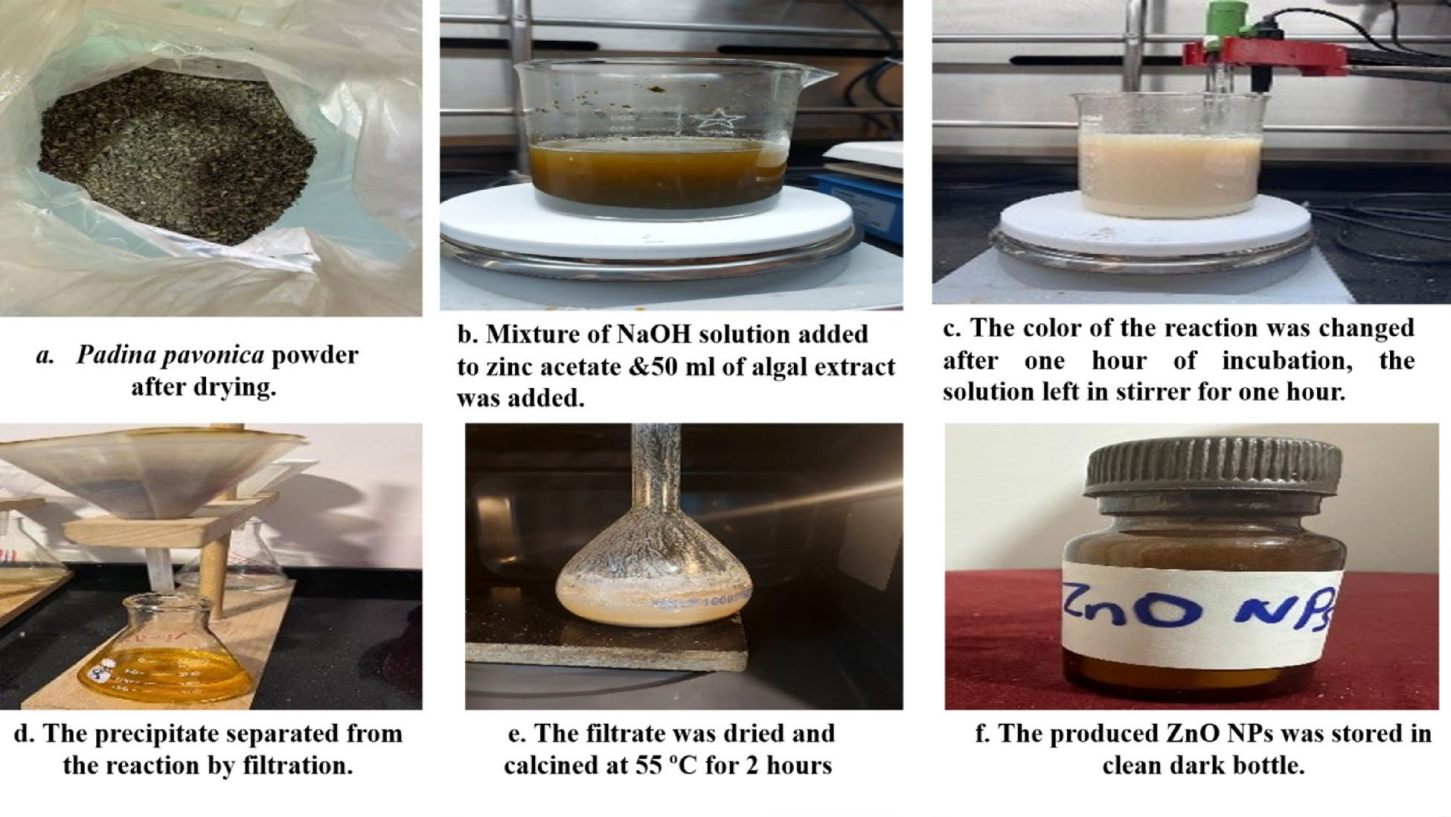
Green Synthesis of ZnO NP_s_ by using aqueous *Padina pavonica* extract.

Weigh five grams of Zinc acetate [Zn(O_2_CCH_3_)_2_(H_2_O)_2_], dissolve in 50 ml DDW and keep in stirrer for 30 min respectively. Add 20 mL of NaOH solution slowly to the Zinc acetate solution, and 50 mL of *Padina pavonica*. The pH of the reaction mixture was maintained at pH = 10 with the help of 0.2 M NaOH solution. The color of the reaction mixture was changed from white to reddish brown after 1 h of incubation time. The solution was left in stirrer for 1 h. The precipitate was separated from the reaction solution by filtration and washing with H_2_O. The filtrate was dried and calcined at 550 °C for two hours^20^ and shown in Fig. 1.

### Chemical synthesis of ZnO QD_s_

**Fig. 2:**
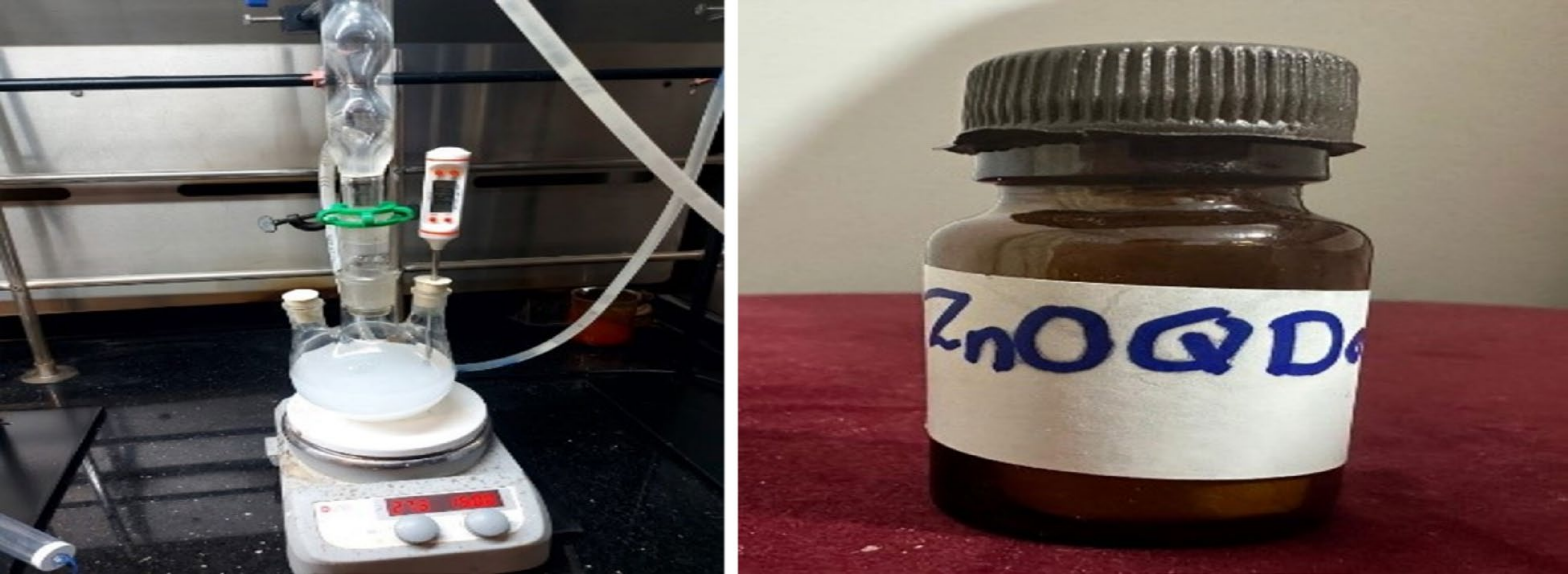
Chemical Synthesis of ZnO QD_s_ by a precipitation method.

As shown in Fig. 2, the chemical synthesis of ZnO QDs was done according to the method described by^21^. The first step is to synthesis ZnO QD_s_ at 60 °C, 140 mmol KOH was dissolved in 80 mL ethanol, and then reduced temperature to 4 °C. 100 mmol Zn (Ac)_2_·2H_2_O was put into a flask with a 600 mL ethanol solution and kept stirring at 78 °C until dissolved. The KOH solution was added to the Zn(Ac)_2_·2H_2_O solution slowly, and reacted for 30 min to result in a ZnO QD_s_ solution. Then the unmodified ZnO QD_s_ (U-ZnO QD_s_) appeared by mixing the ZnO QD_s_ solution with CH_3_(CH_2_)_4_CH_3_ at 1:2 volume ratio. The second step of modifying ZnO QD_s_ was achieved by adding a mixed solution of 1.6 mL APTES and 6 mL deionized water dropwise to the ZnO QDs solution. After this, silane surface modified ZnO QD_s_ (M-ZnO QD_s_) gradually all precipitated out. Finally, the precipitate was centrifuged by a centrifuge (6000 rpm, 2 min) and the precipitate was washed fifth with ethanol to remove the unreacted precursors. The powders of the U-ZnO QD_s_ and M-ZnO QD_s_ were achieved by drying (60 °C, 6 h) the washed sediment and dissolved into water for further applications^22^.

### Characterization of zinc oxide quantum dots [ZnO QD_s_] and zinc oxide nanoparticles [ZnO NP_s_

Both Zinc oxide quantum dot (ZnO QD_s_) and Zinc oxide NP_s_ (ZnO NP_s_) were characterized using five advanced devices. The UV absorbance in the range of 200–700 nm range was characterized using a Shimatzu UV −2600 spectrophotometer using BaSO_4_ as reference^23^. The [HRTEM, Jeol, JEM-2100 plus] at an accelerating voltage of 200KV was used to assess the size of both. The active groups in both NMs were identified using FTIR analysis at a range of 4000–400 cm^−1^. FTIR spectroscopy [Nicolet IS50, Thermo Electron Corporation, Waltham, MA]^24^. The elemental composition of both nanomaterials was determined using EDX and the nature of both were detected by Bruker X-ray Diffractometer at 40 kV and 30 mA with a wavelength equal to 1.5406 Aº XRD^24^.

### Photocatalytic study

The potential application of ZnO NP_s_ and ZnO QD_s_ towards dye removal from wastewaters was evaluated in heterogenous photocatalysis route. MB, Rh 6G and Fluorescein were used to test the photocatalytic activity of the ZnO nanoparticles and quantum dots. Photocatalysis reaction was carried out in a homemade photoreactor equipped with a Xenon arc lamp power supply (Model A 6000, USA) and a fully automatic dual-range AC voltage regulator (50–130 V) (Model ST 3000 W, USA). The lamp is positioned in the front of beaker. The distance between the lamp and glass beaker is 25 cm. For each experiment, take 0.15 g of ZnO NP_s_ to dispersed in 50 ml aqueous solution of MB and Rh 6G and 0.15 g of ZnO QD_s_ to dispersed in 50 ml aqueous solution Fluorescein and Rh 6G with a constant concentration 0.015 g/L. Prior to UV illumination, the suspension was magnetically stirred in the dark for 60 min in order to obtain proper homogeneity of the mixture as well as to reach the absorption equilibrium. At fixed irradiation times (20, 40 and 60 min), samples were finally analyzed by Ultraviolet-Visible absorption spectroscopy (Spectro UV-Vis 2800, United States). The percentage of photocatalytic degradation was calculated using the following equation:

1 $$\:\text{P}\text{e}\text{r}\text{c}\text{e}\text{n}\text{t}\text{a}\text{g}\text{e}\:\text{P}\text{h}\text{o}\text{t}\text{o}\text{d}\text{e}\text{g}\text{r}\text{a}\text{d}\text{a}\text{t}\text{i}\text{o}\text{n}=\frac{\text{A}\text{o}-\text{A}}{\text{A}\text{o}}\times\:100\%$$

The rate constant of the degradation, K, was obtained from first-order plot according to the equation: ln (A_o_/A) = kt, where A_o_ is the initial absorbance of dye/ZnO nanomaterial and A is the absorbance of dye/ZnO after UV light irradiation^25^.

### Anticancer application

Measurement of Cytotoxicity Test (MTT Assay).

A comparative study on ZnO NP_s_ and ZnO QD_s_ nanotoxicity was evaluated on cancer cells using the MTT assay following Mosmann^26^. This assay is a sensitive, quantitative, and reliable colorimetric method that measures cell viability and proliferation. For the chemosensitivity test, exponentially growing cells were harvested, counted, and inoculated (at the appropriate concentrations in a volume of 100 L) into 96-well microtiter plates; 8 replicates were prepared for each dose. U-bottom microplates were used for suspension-growing cells, whereas flat-bottom microplates were used for plastic-adherent cell cultures. Immediately or 24 h after cell seeding, 10 L of different dilutions of drugs, prepared 10 more concentrated than requested, was added to each well. The MTT assay was performed after different incubation times at 37 °C in a humidified 5% CO_2_ atmospheres. MTT (Sigma, St. Louis, MO, USA) was dissolved at a concentration of 5 mg/ml in Hank’s salt solution and filtered with a 0.45 filter (in order to avoid MTT aggregates). Ten microliters of the MTT solution were added to each well and the control wells without cells. Additional controls consisted of media alone with no cells, with or without the various drugs. After 4–6 h of incubation, microtiter plates were centrifuged at 2000 rpm for 10 min; the medium was removed, and 100 L of DMSO was added to each well. After thorough mixing with a mechanical plate mixer, the absorbance of the wells was read in a scanning well microculture plate reader at the test and reference wavelengths of 550 and 620 nm, respectively, which are approximately the peak and lowest MTT wavelengths of absorption required to avoid quenching from the growth medium, in particular, phenol red. Absorbance values from all wells were corrected against these control absorbance levels. The ID50 was defined as the concentration of drug that produced a 50% reduction in absorbance compared with untreated control cells^27^.

The methodology used to calculate the viability percentage %under Cytotoxicity test was as following:

The IC50 was calculated using masterplex 2010 software. The viability % was calculated using the following equation:

Viability % = mean OD of test dilution divided by the mean OD of control multiplied by 100. This according to Ahmed et al., (2021)^53^.

## Results and discussion

### X-ray diffraction

As indexed and carried out the X-ray diffraction in the paper under publication [**Abdelfatah Salah Soror**,** et al. 2025**]. The exact nature of the formed ZnO NP_s_ & ZnO QD_s_ may be deduced from the XRD spectrum of sample. XRD confirmed that the crystalline properties of ZnO NPs & ZnO QD_s_. The patterns of XRD spectrum were investigated and evidenced by the peak at 2θ values were the diffraction pattern for ZnO NP_s_ showed some peaks at 2θ values 31.69^o^,34.35^o^,36.17^o^,47.46^o^ and 56.51^o^ corresponding to miller indices [hkl], [100], [002], [101], [102] and [110] respectively. The strong peak was recorded at plane [101] define the structure of ZnO NP_s_. The farther understand the crystalline properties of the prepared ZnO QD_s_, X-ray Diffraction (XRD) was utilized. The pattern of XRD were studded in the range of 2θ values 31.78^o^, 34.45^o^, 36.27^o^, 47.55^o^, 56.61^o^, 62.87^o^, 66.45^o^, 67.94^o^, 69.07^o^, 72.56^o^ and 76.92^o^ corresponding to miller indices [hkl], [100], [002], [101], [102]. [110], [103], [200], [112], [201], [004] and [202]. It could be stated that the strong peak was found at plane (101). The Bruker X-ray Diffractometers confirm the crystalline character of ZnO QD_s_. Bragg’s law is the underlying theory of X-ray diffraction^28^. Finally, an X-ray diffraction (XRD) examination verified the crystalline form of ZnO NP_s_ & ZnO QD_s_. The XRD pattern confirmed the presence of crystalline hexagonal wurtzite phase structure in both ZnO NP_s_ and ZnO QD_s_ with average crystallite size in the range 30–40 nm with different reaction times^29^. Using the Debye-Scherrer equation D = Kλ/βCosθ, it is possible to determine the average ZnO NP_s_ & ZnO QD_s_ crystallite size. Where K is Scherrer constant, and its value is 0.94^30^. **λ** is the wavelength of X-ray. β is the full width of full maximum. θ is the Bragg angle. D is nanoparticle crystalline size. The XRD showed the crystallite size of the ZnO NPs to be 35 nm and of the ZnO QD_s_ to be 7.5 nm.

### High Resolution Transmission Electron Microscope (HR-TEM)

As indexed and carried out the HR-TEM spectra in the paper under publication [**Abdelfatah Salah Soror**,** et al. 2025].** Regarding the morphological structure of both ZnO NPs & ZnQ QDs, they were depicted by using HR-TEM. The HR-TEM was used to examine the morphology, size and crystalline properties of both. The size of spherical particles of ZnO NP_s_ was in range of 9–90 nm. While ZnO QDs clearly showed random distribution of homogenous fine spherical shaped particles with overage particle size of 7.6 nm which is finally consistent with calculated average of crystalline size from XRD.

### Energy-Dispersive X-ray (EDX)

As indexed and carried out the EDX spectra in the paper under publication [**Abdelfatah Salah Soror**,** et al. 2025].** The EDX image on the sample powdered of ZnO NP_s_ assured that the main constituent elements are Zn in addition to presence of other elements as carbon and oxygen. EDX shows the qualitative analysis of EDX of ZnO NP_s_ and clarified that the strong peak of Zn at 7.55 (mass%) which recorded at 8.630 (Kev). Also, strong peak of oxygen at 35.09 (mass%) at 0.525(Kev). There were traces of other compounds like carbon which showed in 57.36 (mass%) and at 0.277(Kev). Regarding ZnO QD_s_ also, EDX of quantitative analysis of ZnO QD_s_ shows the main constituent elements is Zn and the strong peak of Zn was recorded at 4.17(mass%) and 8.630 (Kev). Moreover, a strong peak of oxygen was also found at 21.54 (mass%) and 0.525 (Kev). In addition to other compounds like carbon was recorded at 74.29 (mass%) and 0.227(Kev)^31^.

### Fourier Transform Infrared Spectroscopy (FTIR) of ZnO NP_s_ & ZnO QD_s_

As indexed and carried out the FTIR spectra in the paper under publication [**Abdelfatah Salah Soror**,** et al. 2025].** The FTIR technique is utilized to obtain information about functional interactions, various types of interactions, and group bonding. The functional groups in the algal solution that are responsible for reduction, capping, and stabilization are identified using FTIR^32^. FTIR of prepared samples illustrated that eight bands were detected in ZnO NP_s_. According to the FTIR spectrum the tested ZnO NP_s_ were surrounded by a reducing agent. Eight bands were ascribed to active groups in ZnO NP_s_ suspension (668.14, 1362.47, 1418.92, 1507.12, 1616.84, 1733.85, 2143.12 and 2524.49 cm^−1^). These bands were Halo compounds C-Br, Sulfonamide S = O, Alcohols OH, Nitro compound N = O, α, β unsaturated ketone C = O, Ketones C = O, Carbodiimide N = C = N, carboxylic acid COOH. Moreover, Fig.12 showed that six bands were detected in ZnO QD_s_. According to the FTIR spectrum, the tested ZnO QD_s_ were surrounded by a reducing agent. six bands were ascribed to active groups in ZnO QD_s_ suspension (781.9858, 1504.45037, 2091.4528, 2183.8133, 2916.54022 and 3565.1 cm^−1^). These bands were Alkene C = C, Nitro compound N = O, Isothiocyanate N = C = S, Alkene C = C, Alcohol OH and Alcohol OH.

### Photocatalytic Activity (PC) of ZnO NP_s_ dispersed in MB and Rh 6G & ZnO QD_s_ dispersed in fluorescein and Rh 6G dyes

**Fig. 3:**
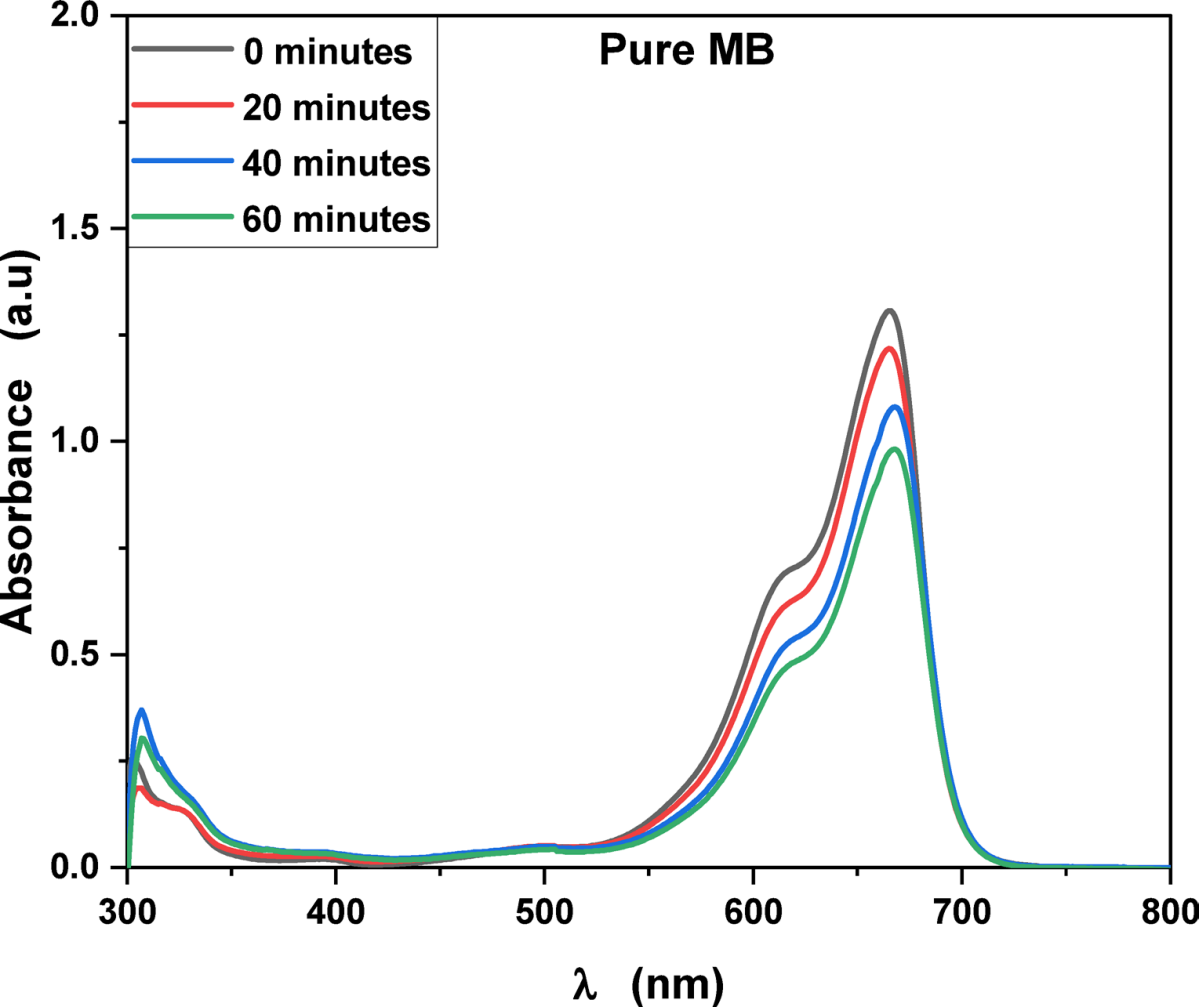
UV-Vis absorbance spectra of pure methylene blue at different irradiation times.

**Fig. 4:**
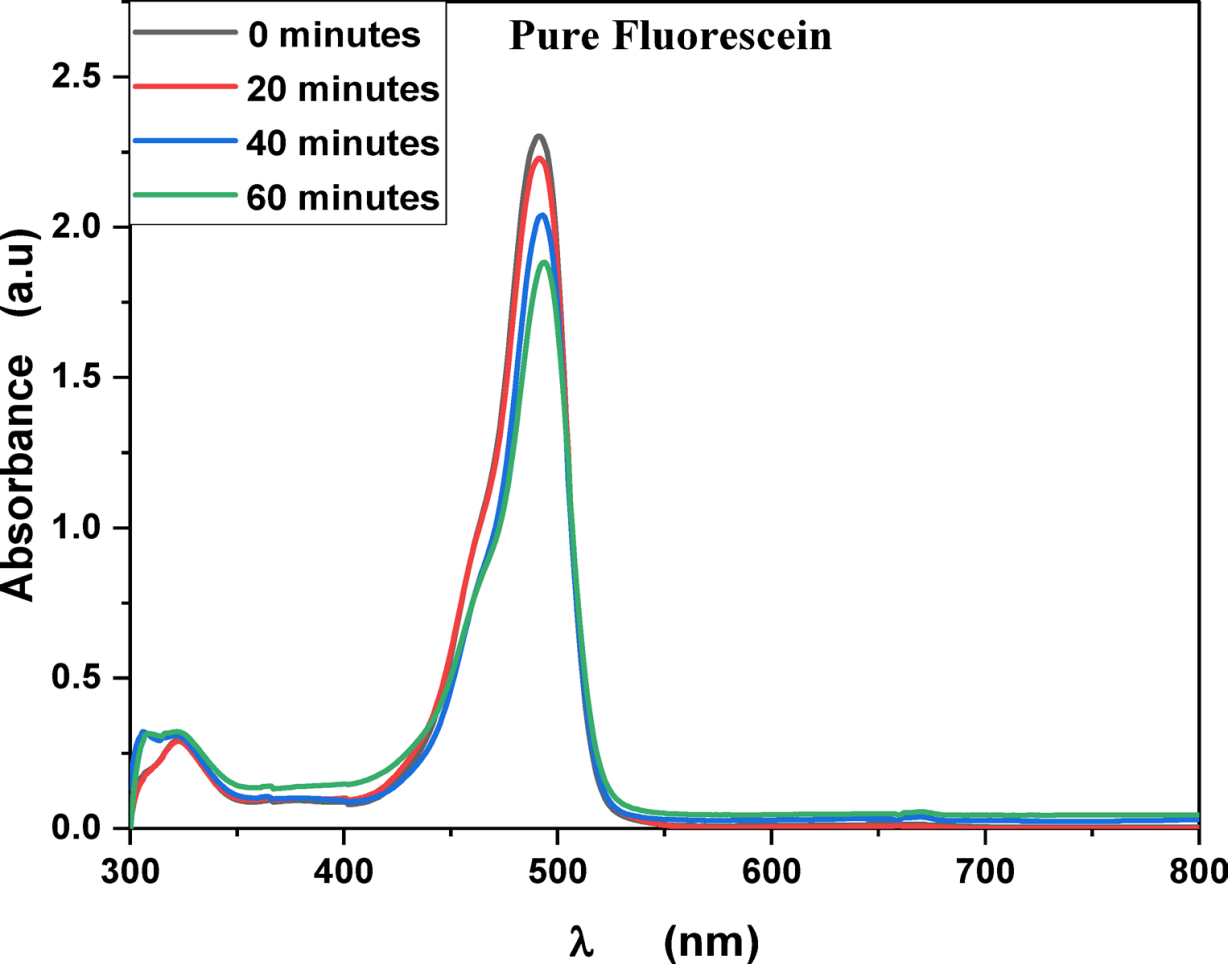
UV-Vis absorbance spectra of pure Fluorescein at different irradiation times.

**Fig. 5:**
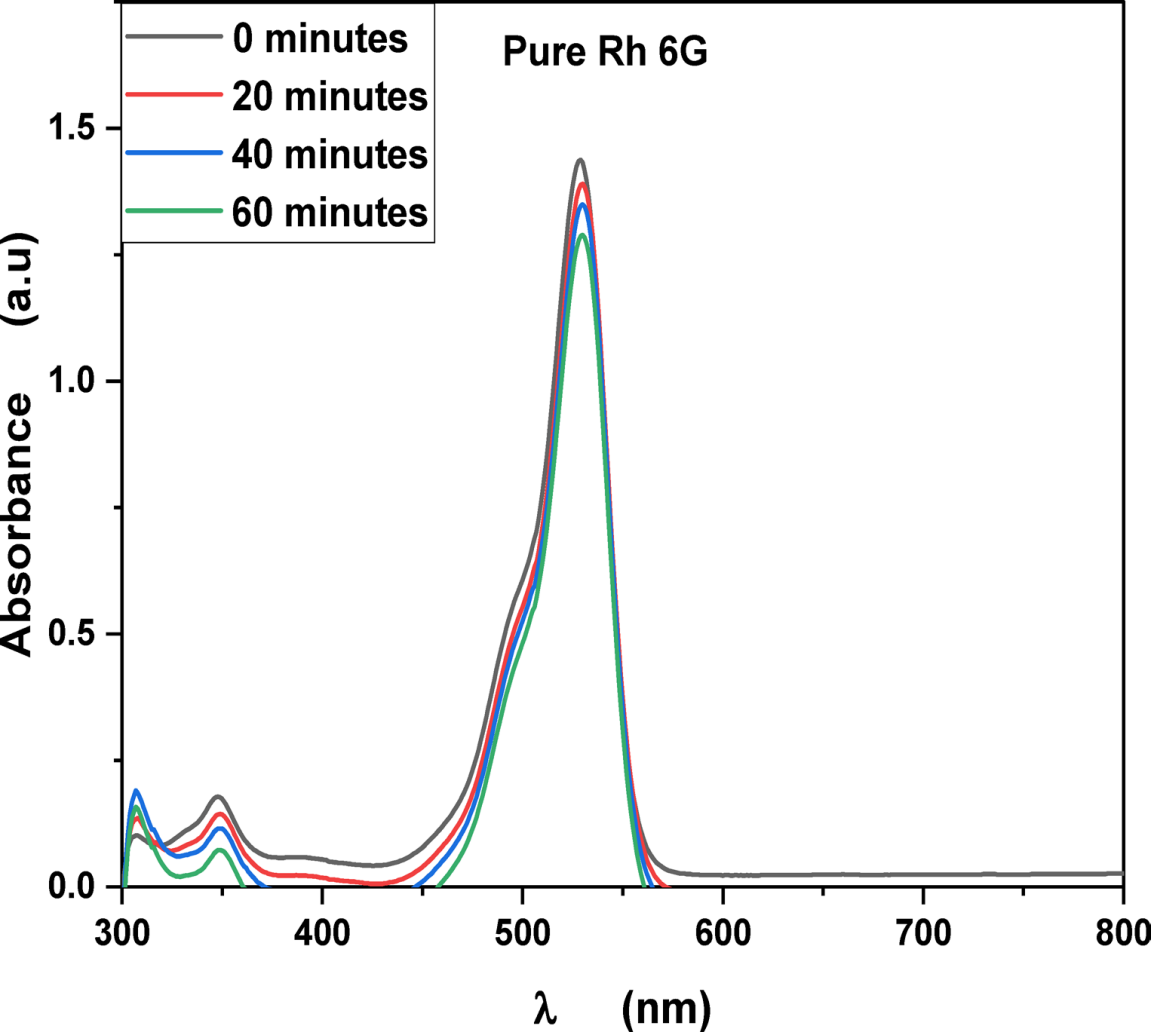
UV-Vis absorbance spectra of pure Rhodamine 6G at different irradiation times.

**Fig. 6:**
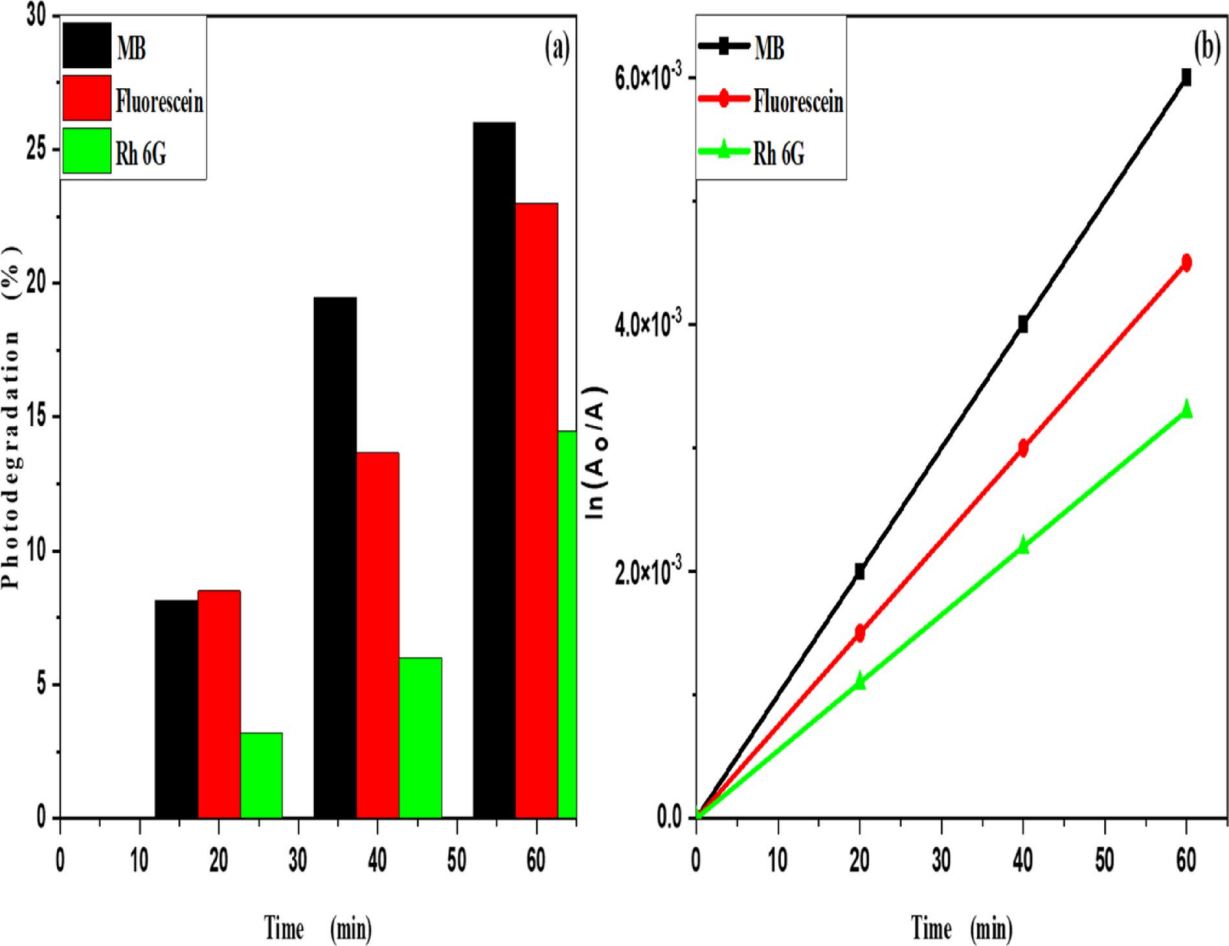
(**a**) Percentage photodegradation and (**b**) Kinetic plot of ln (A_o_/A) vs. irradiation time of pure MB, pure Fluorescein and pure Rhodamine 6G.

**Fig. 7:**
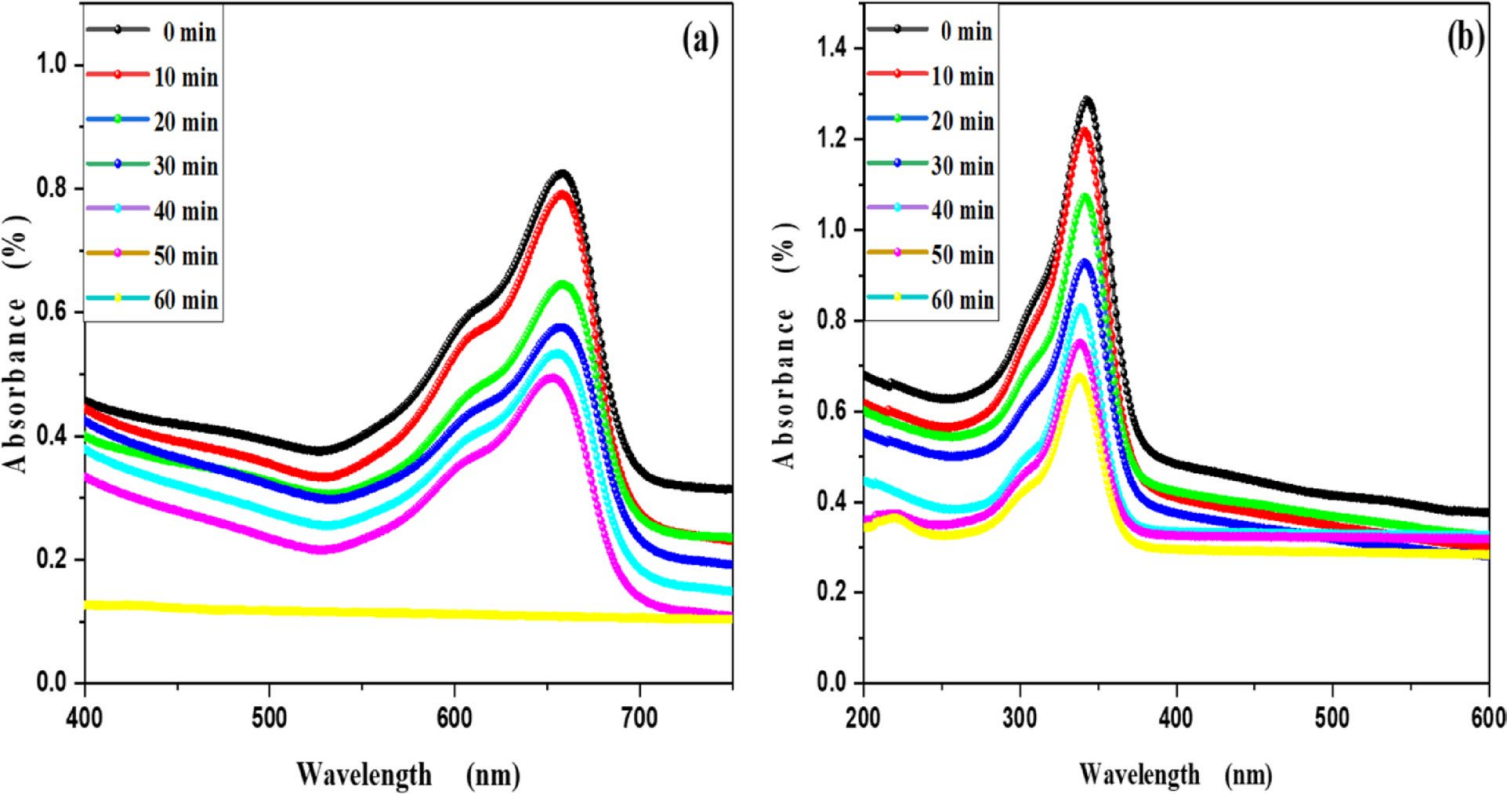
UV-Vis absorbance spectra of (**a**) methylene blue and (**b**) Rhodamine 6G in the presence of the ZnO QD_s_.

MB, Fluorescein and Rh 6G organic dyes, commonly found in fabric wastes, are frequently used as a model pollutant in wastewater treatment to evaluate the catalysts. Fig. 3 shows the optical absorption spectra of pure methylene blue UV light irradiation which show that the optical absorbance decreased from 8.16% at 20 min to 26% at 60 min. Fig. 4 shows the optical absorbance of pure Fluorescein under UV irradiation decreased from 8.51% at 20 min to 22.99% at 60 min. Fig. 5 shows that the optical absorbance of the pure Rh 6G under UV irradiation decreased from 3.14% at 20 min to 14.48% at 60 min. As illustrated in Fig. 6a the photodegradation of methylene blue is higher than photodegradation of Rh 6G and Fluorescein, while Fig. 6b shows the kinetic rates (K) of pure MB, pure Fluorescein and pure Rh 6G dyes adhere to a pseudo-first-order rate equation, i.e., ln(A_0_/A) = Kt that are shown in Table 1. Figure 7a,b shows the optical absorption spectra of the of MB and Rh 6G under UV light irradiation in the presence of ZnO quantum dots as a photocatalyst and we observed that by increasing irradiation time from 0 min to 60 min the optical absorbance of pollutants dye was decreased. So, when we had compared the photodegradation of Rh 6G dye with and without ZnO QD_s_, we found that the Rh 6G dye in the presence of the ZnO QD_s_ shows the absorbance degradation peak between 300 nm and 400 nm, whereas pure Rh 6G shows absorbance peak between 500 nm and 600 nm. So, there is a shifting in absorption spectra to a lower wavelength.

**Fig. 8:**
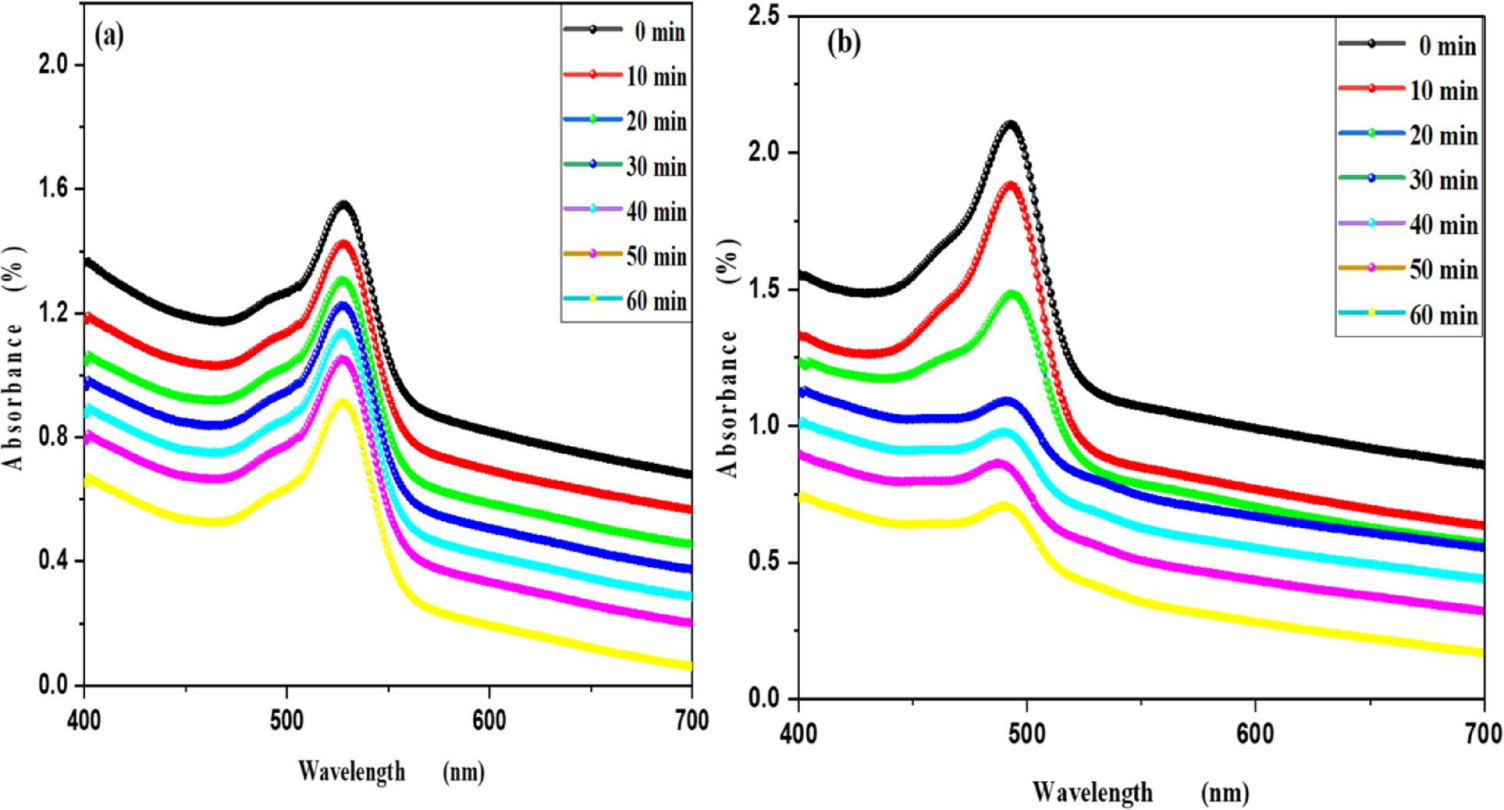
UV-Vis absorbance spectra of (**a**) Rhodamine 6G and (**b**)Fluorescein in the presence of the ZnO NP_s_.

Figure 8a,b shows the optical absorption spectra of the degradation of Rh 6G and Fluorescein dyes under UV light irradiation in the presence of ZnO nanoparticles as a photocatalyst. By increasing irradiation time from 0 min to 60 min, it is clear that the absorbance intensity at 533 nm decrease for Rh 6G dye, the absorbance intensity at 690 nm decrease for Fluorescein dye and confirms that ZnO QD_s_ are acting as photocatalysis for the degradation of Rh 6G and Fluorescein dyes.

**Fig. 9:**
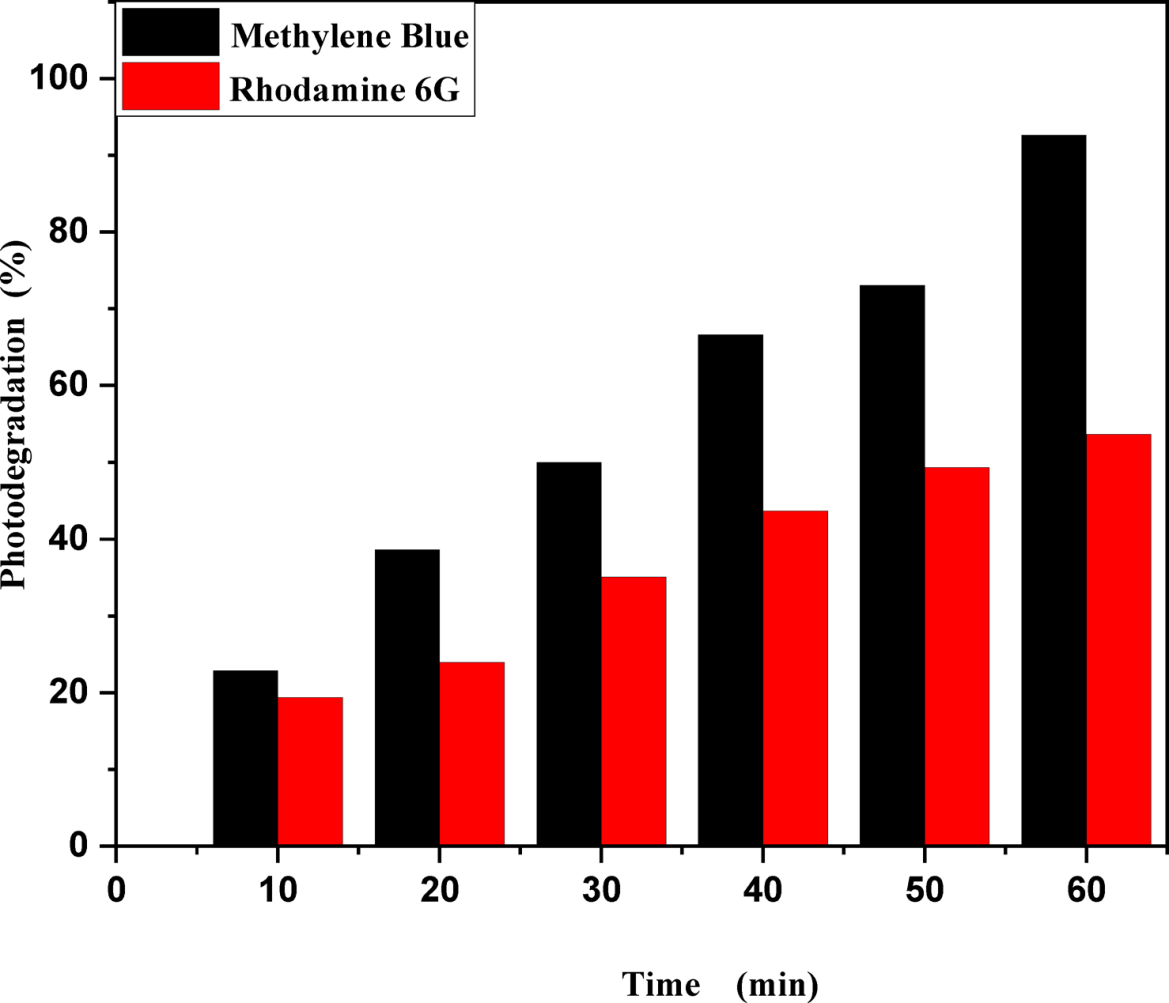
Percentage photodegradation of methylene blue and Rhodamine 6G vs. irradiation time in the presence of the ZnO QD_s_.

**Fig. 10:**
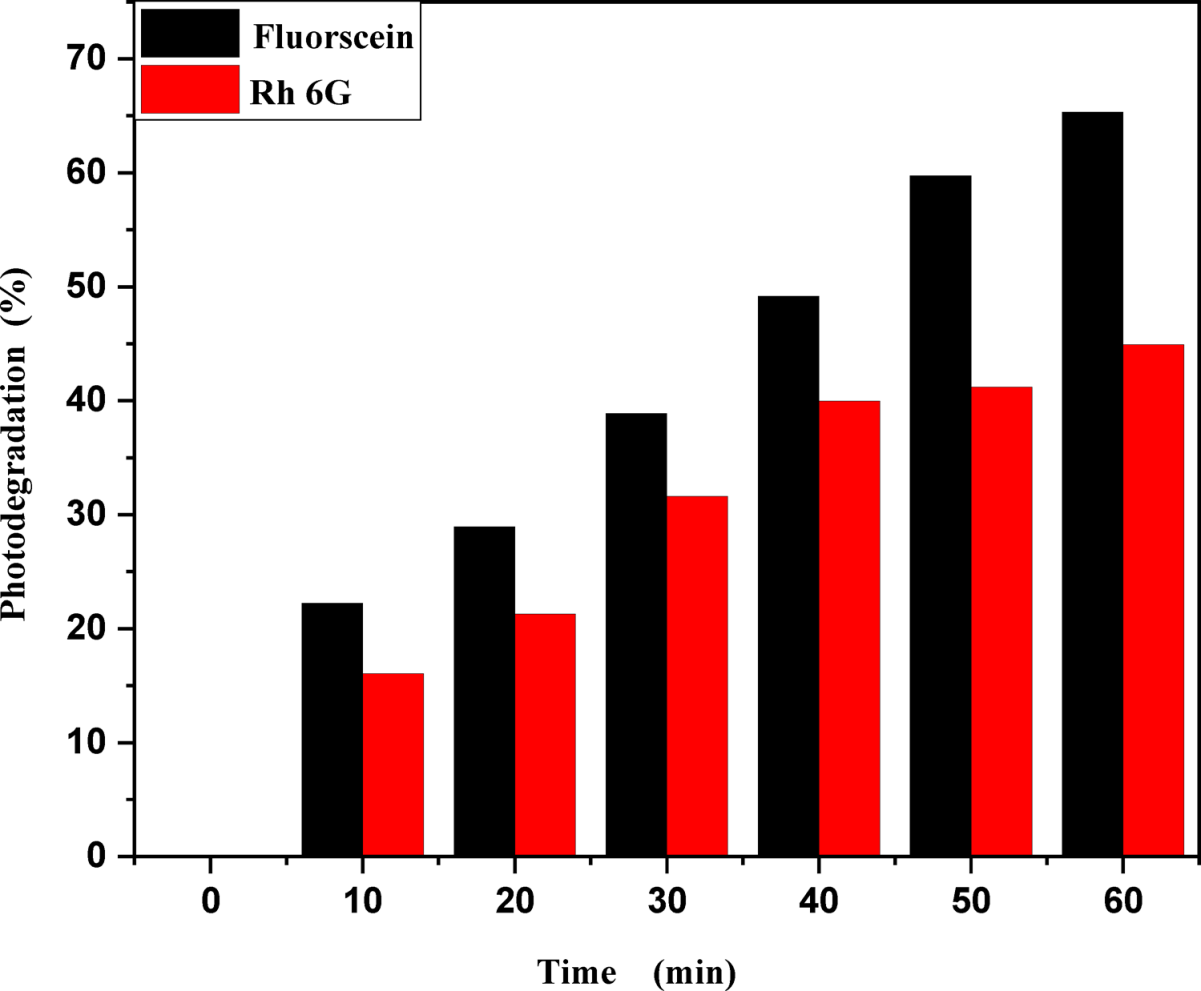
Percentage photodegradation of Rhodamine 6G and Fluorescein vs. irradiation time in the presence of the ZnO NP_s_.

Figure 9a,b shows the photodegradation percentage of MB and Rh 6G in the presence of ZnO quantum dots under UV light irradiation. Where by increasing UV irradiation exposure time, the photodegradation percentage of MB and Rh 6G increasing and its values at different exposure times shown in Table 2. Figure 10a,b shows the photodegradation percentage of Rh 6G and Fluorescein in the presence of ZnO nanoparticles under UV light irradiation. Where by increasing UV irradiation exposure time, the photodegradation percentage increasing for Rh 6G and Fluorescein and its values at different exposure times shown in Table 2.

**Fig. 11:**
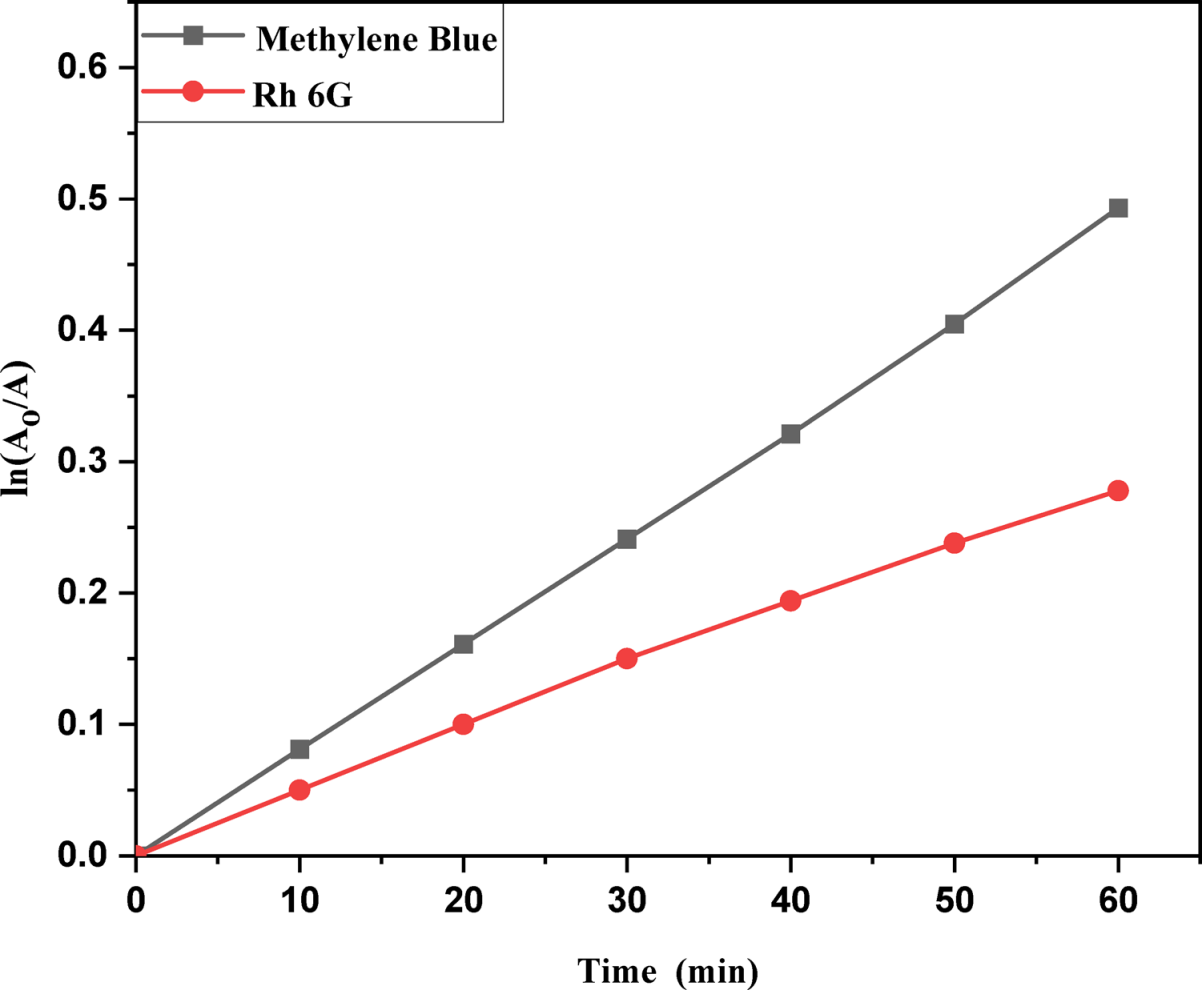
Kinetic plot of ln (A_o_/A) vs. irradiation time of methylene blue and Rhodamine 6G in the presence of the ZnO QD_s_.

**Fig. 12:**
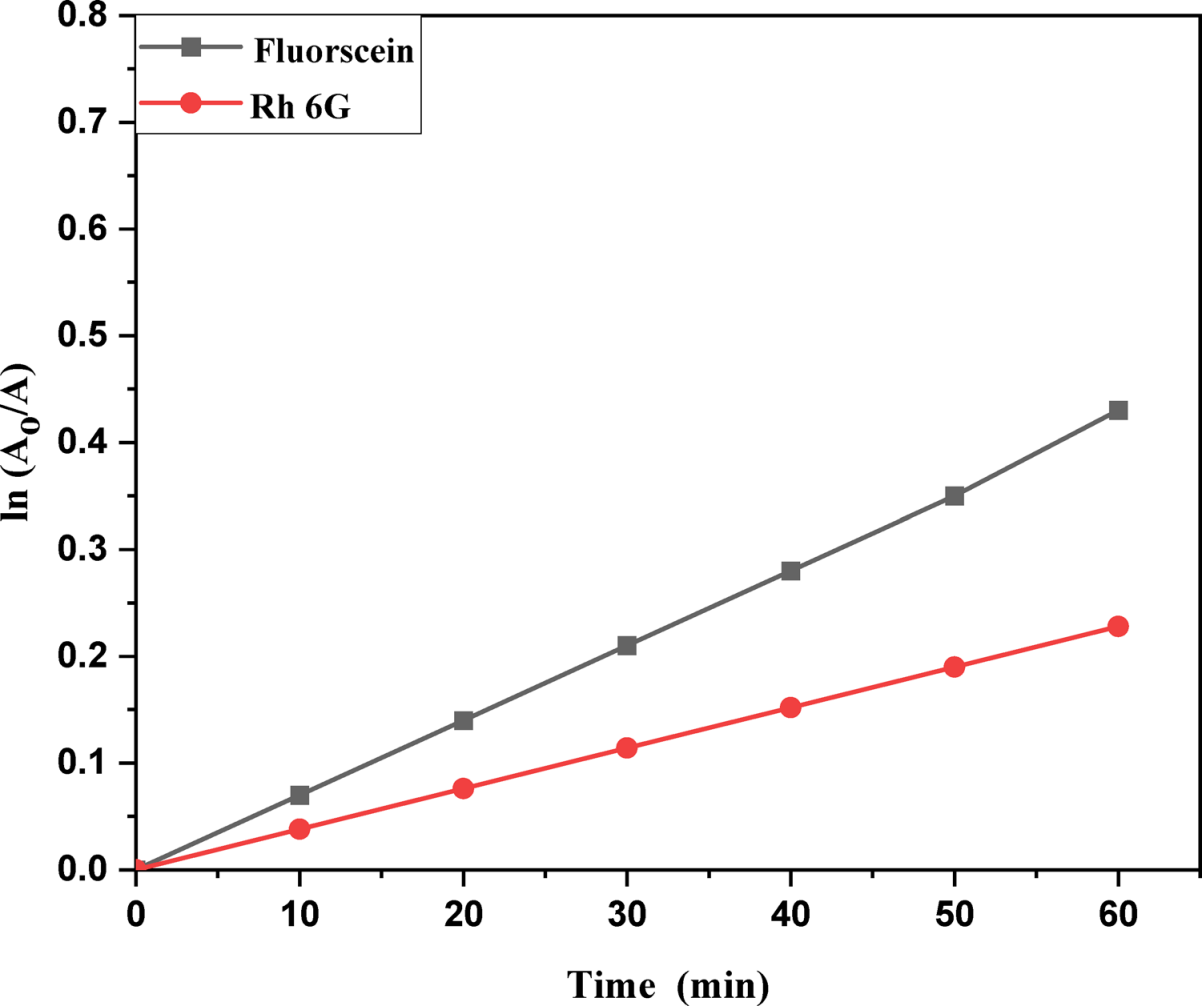
Kinetic plot of ln (A_o_/A) vs. irradiation time of Rhodamine 6G and Fluorescein in the presence of the ZnO NP_s_.

The kinetic study for the degradation of MB, Rh 6G and Fluorescein dyes were studied using the Langmuir-Hinshelwood kinetic model: ln (A_o_/A) = kt. A plot of ln (A_o_/A) versus t is shown in Fig. 11a,b for MB and Rh 6G with ZnO QD_s_ and Fig. 12a,b) for Rh 6G and Fluorescein with ZnO NP_s_ and the rate constants are shown in Table 2. Photocatalytic activity occurs because of the interaction of photocatalyst and UV light irradiation, which are believed to be the main species responsible for the oxidation. Other active species such as holes, electrons and superoxide act as oxidant species for the degradation of dyes^33^.

In summary, ZnO quantum dots (QD_s_) exhibit superior photocatalytic activity compared to ZnO nanoparticles (NP_s_) for dye degradation due to their enhanced light absorption and efficient charge separation. Specifically, ZnO QD_s_ with their smaller size and quantum confinement can offer higher quantum efficiency. The quantum confinement effect in QDs leads to larger band gap resulting in better light absorption. Additionally, the smaller size of ZnO QDs facilitates the separation of photogenerated electron-hole pairs, which are crucial for the degradation process.

**Table 1:** Percentage photodegradation and kinetic rates (K) for pure MB, pure Rh 6G and pure fluorescein dyes under UV irradiation.

Dye	Rate constant (min^−1^) ± 0.0239			Degradation Percentage (%)		
	20	40	60	20	40	60
Methylene Blue	0.0032	0.0043	0.0045	30.93	43.85	58.79
Fluorescein	0.0017	0.0029	0.0033	26.77	38.64	50.23
Rh 6G	0.0014	0.0015	0.0017	13.65	16.22	22.56

**Table 2:** Photocatalytic degradation percentage and rate constant for MB and Rh 6G dyes in the presence of ZnO QD_s_, Rh 6G and fluorescein dyes for ZnO NP_s_.

Catalyst	Dye	Rate Constant (min-1 ± 0.022)						Photodegradation (%)					
		Time (minutes)											
		10	20	30	40	50	60	10	20	30	40	50	60
ZnO QDs	Methylene Blue	0.0012	0.0028	0.0052	0.0065	0.0089	0.0099	22.86	38.63	49.97	66.61	73.08	92.64
	Rh 6G	0.00041	0.00067	0.0037	0.0043	0.0049	0.0053	19.39	23.96	35.09	43.62	49.30	53.62
ZnO NPs	Fluorescein	0.0045	0.0055	0.0068	0.0081	0.0087	0.0093	22.25	28.93	38.88	49.18	59.76	61.32
	Rh 6G	0.00035	0.00042	0.00066	0.0056	0.0089	0.0187	16.06	21.27	31.62	39.96	41.19	44.95

### Optical band gap of MB, Rh 6G and fluorescein in the presence of ZnO QD_s_ and ZnO NP_s_ at different irradiation time

In order to determine the optical band gap, we used the Tauc formula as shown in Eq. 1, which is as follows:

2 $$\left( {\alpha hv} \right){\text{ }} = A{\text{ }}\left( {h\upsilon {\text{ }} - {\text{ }}Eg} \right)^{n}$$

where hυ is the photon energy, E_g_ is the energy gap, and n is the quality of the transitions. For a direct transition, the term n is used as 2, whereas for an indirect transition, it is ½. Plotting (αhυ)^1/n^ versus photon energy and tangent-drawing the curve that meets the energy axis at α = 0 are the methods used to examine the optical bandgap energy.

**Fig. 13:**
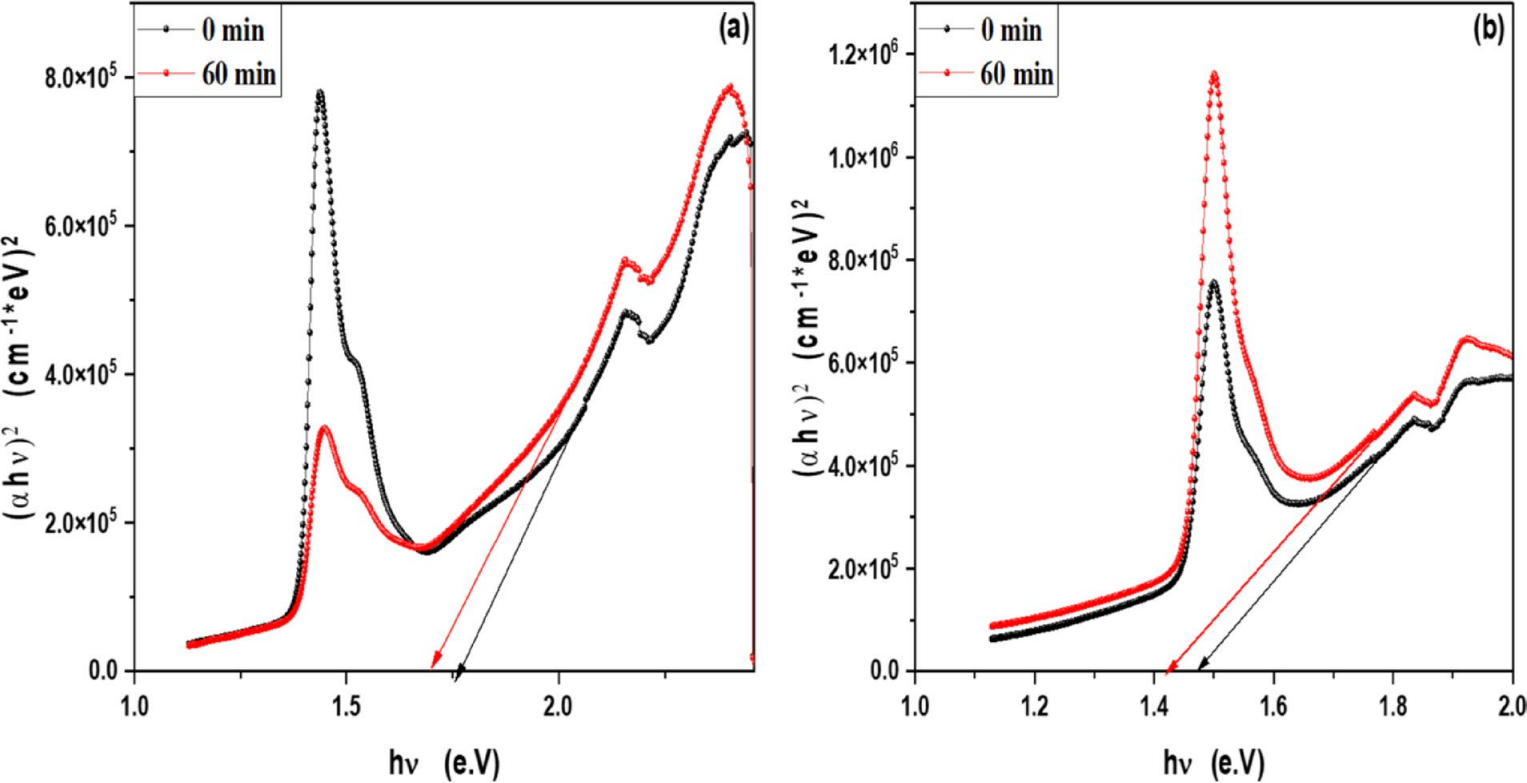
Plot of (αhʋ)^2^ versus hʋ for ZnO QD_s_ in the presence of (**a**) MB dye and (**b**) Rhodamine 6G before and after exposure to UV light irradiation for direct transition.

**Fig. 14:**
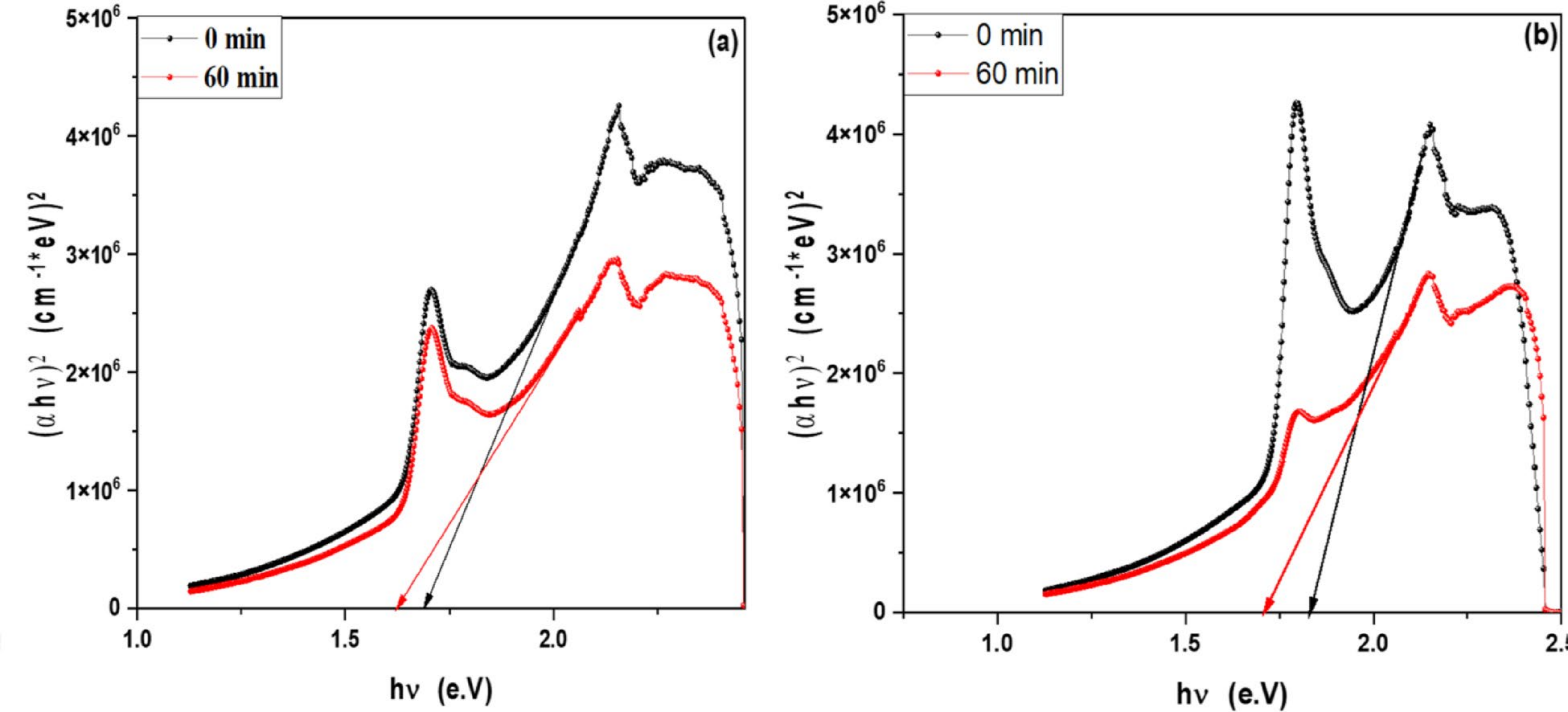
Plot of (αhʋ)^2^ versus hʋ for ZnO NP_s_ in the presence of (**a**) Rhodamine 6G and (**b**) Fluorescein before and after exposure to UV light irradiation for direct transition.

**Fig. 15:**
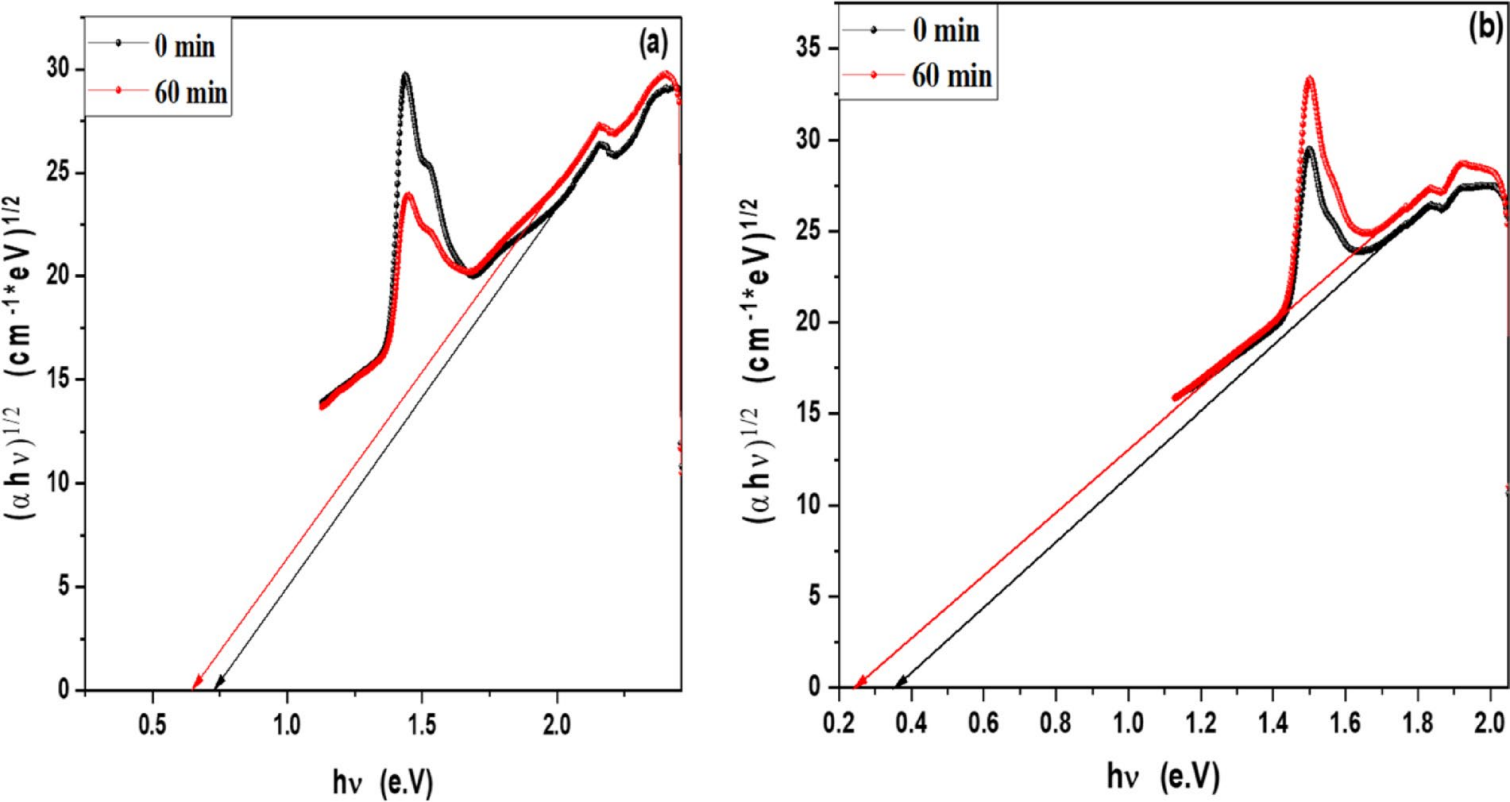
Plot of (αhʋ)^1/2^ versus hʋ for ZnO QD_s_ in the presence of (**a**) MB dye and (**b**) Rhodamine 6G before and after exposure to UV light irradiation for indirect transition.

**Fig. 16:**
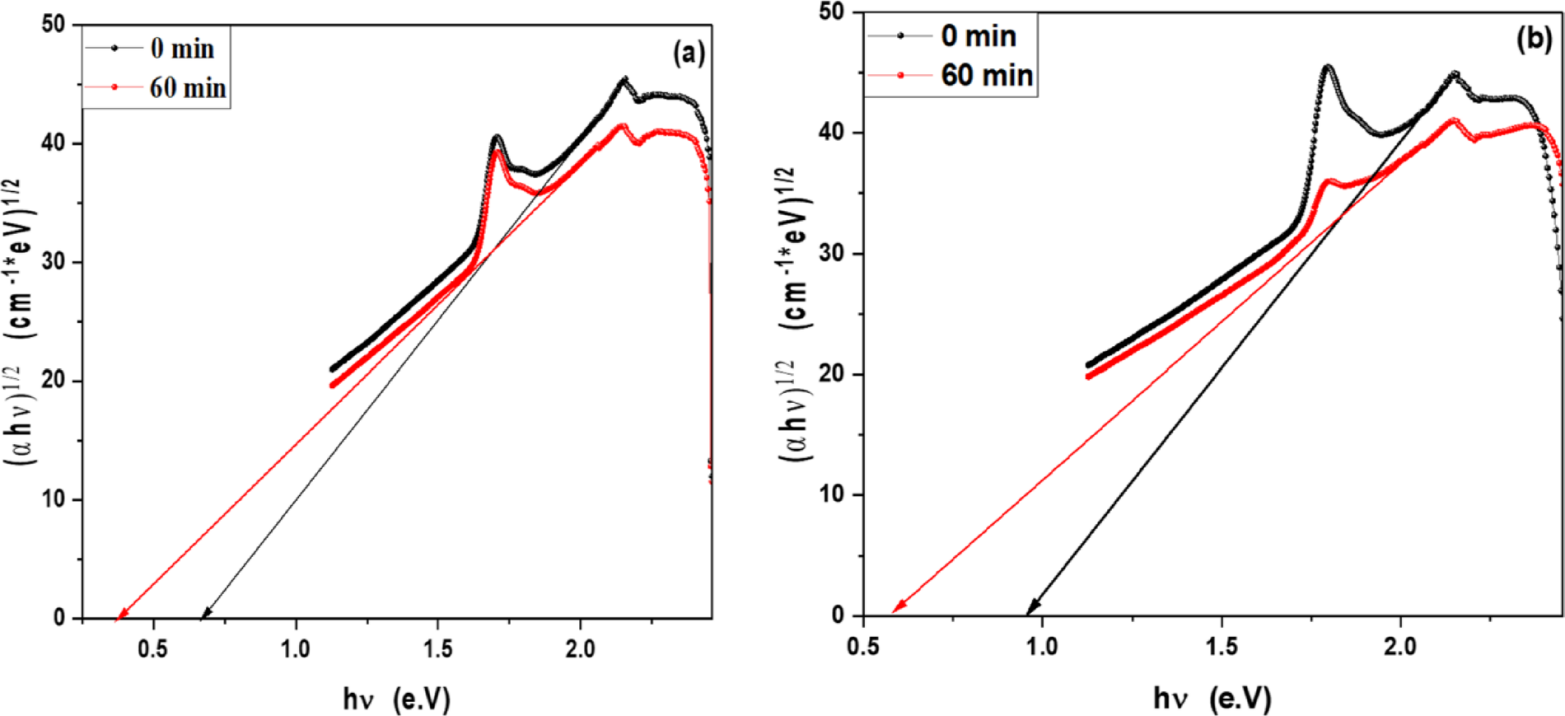
Plot of (αhʋ)^1/2^ versus hʋ for ZnO NP_s_ in the presence of (**a**) Rhodamine 6G and (**b**) Fluorescein before and after exposure to UV light irradiation for indirect transition.

For direct permitted transition, Fig. 13a,b displays the Tauc plot of MB and Rh 6G dye in the presence of ZnO QD_s_. Which shows that the energy gap of ZnO QD_s_ decreasing by increasing irradiation exposure time in both MB and Rh 6G dye. Figure 14a,b displays the Tauc plot for Rh 6G and Fluorescein dye in the presence of ZnO NP_s_ that have a direct allowed transition. Also, Figs. 15a,b and 16a,b shows the Tauc plot for indirect allowed transition for MB and Rh 6G dye in the presence of ZnO QD_s_ and Rh 6G and Fluorescein dye in the presence of ZnO NP_s_ respectively and the values of optical band gap energy of samples shown in Table 3.

**Table 3:** Optical band gap energy for MB and Rh 6G dyes in the presence of ZnO QD_s_ and for Rh 6G and fluorescein dyes in the presence of ZnO NP_s_ before and after irradiation.

Catalyst	Dyes	Exposure Time (min)	E_g_ for Direct transition	E_g_ for indirect transition
ZnO QD_s_	Methylene Blue	0 min	1.77 eV	0.73 eV
		60 min	1.69 eV	0.65 eV
	Rhodamine 6G	0 min	1.47 eV	0.35 eV
		60 min	1.42 eV	0.25 eV
ZnO NP_s_	Rhodamine 6G	0 min	1.69 eV	0.67 eV
		60 min	1.62 eV	0.38 eV
	Fluorescein	0 min	1.83 eV	0.95 eV
		60 min	1.71 eV	0.58 eV

### Anticancer activity of nanomaterials

**Fig. 17:**
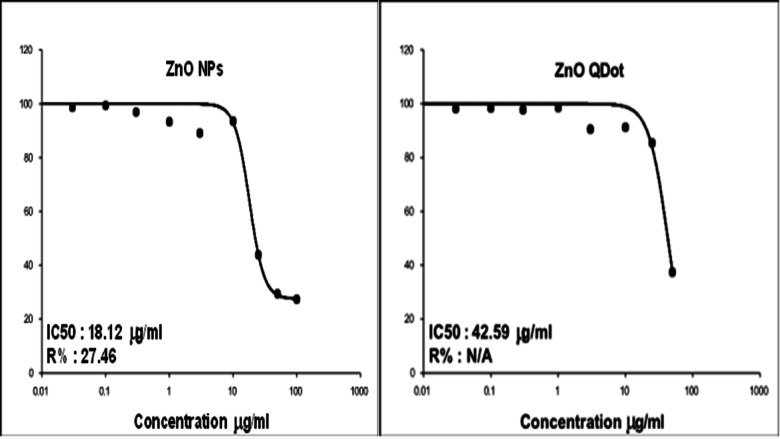
Dose response curve of ZnO NP_s_ fabricated by *padina pavonica* extract and chemically synthesized ZnO QD_s_ against breast ductal carcinoma (T-47D).

**Fig. 18:**
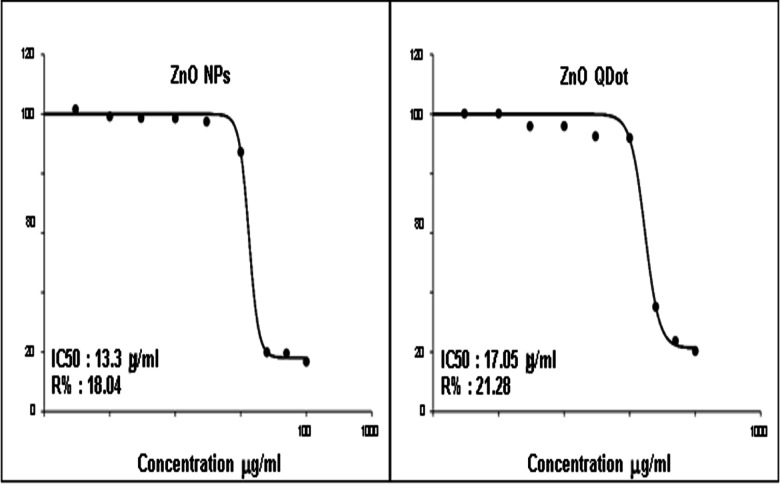
Dose response curve of ZnO NP_s_ fabricated by *padina pavonica* extract and chemically synthesized ZnO QD_s_ against prostate cancer cell (DU-145).

**Fig. 19:**
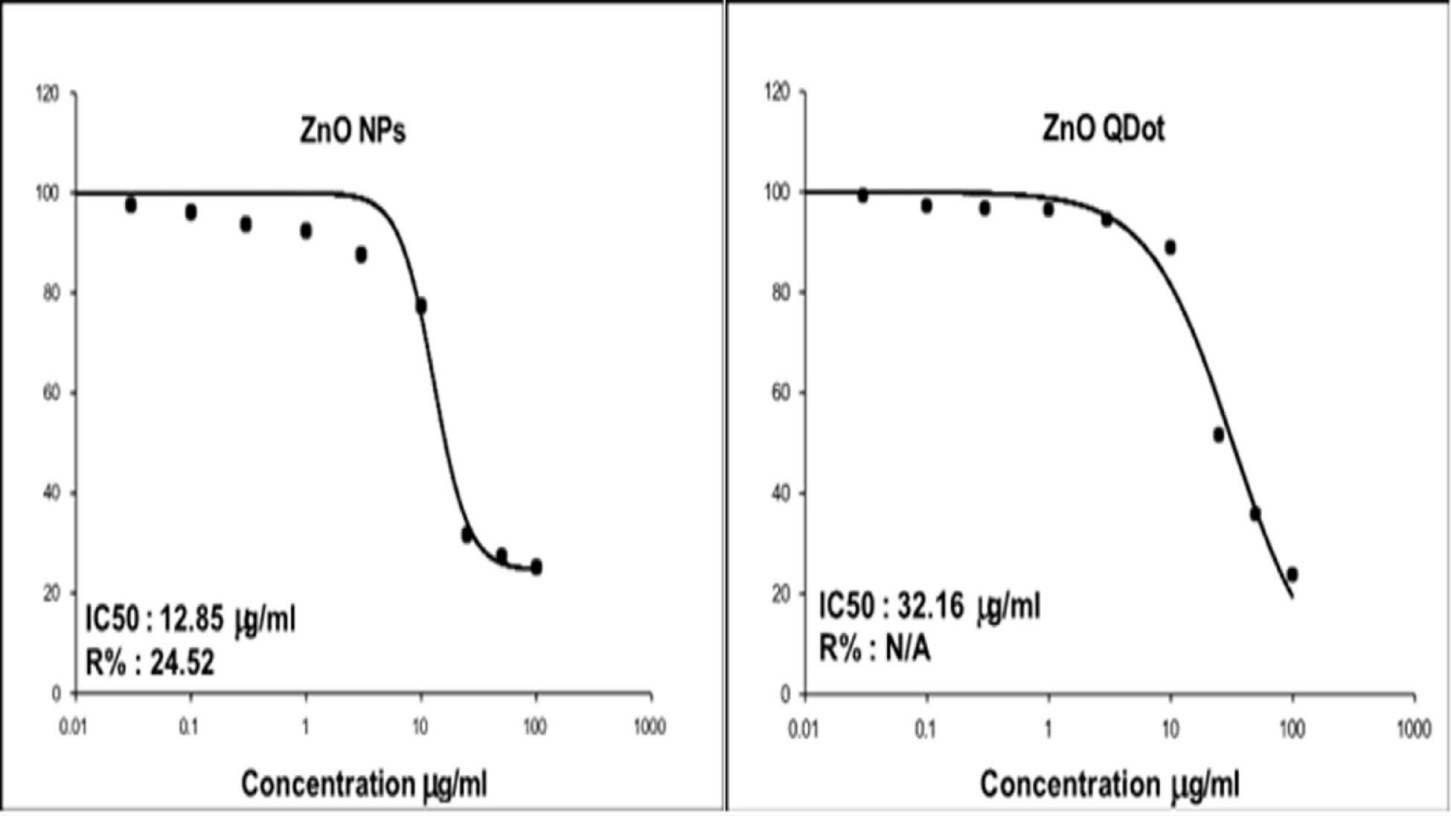
Dose response curve of ZnO NP_s_ fabricated by *padina pavonica* extract and chemically synthesized ZnO QDs against HSF: Human skin fibroblast.

In the present study, MTT assay was conducted to evaluate the toxicity of ZnO NP_s_ fabricated by *padina pavonica* extract and ZnO QDs chemically synthesized against two cell lines, breast ductal carcinoma (T-47D) as shown in Fig. 17, prostate cancer (DU-145) as ahown if Fig. 18 compared to human skin fibroblast (HSF) as shown in Fig. 19, which represent normal cell line^34^. The MTT test depends on the capacity of mitochondrial lactate dehydrogenase (LDH) in living cell to transform the water-soluble substrate, MTT, into water insoluble dark blue formazan. Dimethyl sulfoxide is used as a solubilization to transform the insoluble purple formazan result into colored solution. Its absorbance was measured spectrophotometrically at a wave length of 500–600 nm, according to^35^. Ten concentrations of ZnO NP_s_ and ZnO QD_s_ were prepared and monitored for their effects on the viability and proliferation cancer cell lines regarding their metabolic reduction potency. It was found that ZnO NP_s_ exhibited cytotoxic effect against both normal and cancer cell line in a dose dependent manner. As shown in Figs. 17, 18 and 19, the dramatic effect of ZnO NP_s_ on the viability of treated cell Lins shown in Tables 6,9 and 12 clarified that as the concentrations of ZnO NP_s_ increased a significant reduction in cell viability was observed. However, the highest percentages cell viability was recorded at the lowest ZnO NP_s_ concentration [0.03 µg/ml], since they were 99.33,100 and 97.555% in T-47D, DU-145 and HSF respectively. On the other hand, the lower cell viability was recorded at the highest concentration of ZnO NP_s_ 100 µg/ml, where they were 27.32,16.72 and 25.14 in T-47D, DU-145 and HSF respectively. These results were confirmed by the percentages of inhibitions, since they recorded 72.68,83.28 and 74.86 respectively. Cytotoxicity test of ZnO QDs chemically synthesized against Du-145 Prostatate cancer cell shows at low concentration [0.03 µg/ml], the viability % were 100% and 99.12% in HSF Human skin fibroblast and 98.14% in T-47D breast ductal carcinoma. On the other hand, the lower cell viability was recorded at the highest ZnO QDs concentration [100 µg/ml], where they recorded 37.37,20.28 and 23.6%in the previously mentioned three cell lines. It’s worth mentioned that these results were confirmed by the percentages of inhibition were shown in Tables 5,6, 8, 9, 11 and 12. Moreover, according to IC50, in ZnO NP_s_ as shown in Tables 4, 7 and 10.

As observed in Figs. 17, 18 and 19, the three cell lines can be arranged ascendingly as HSF, DU-145 and T-47D where the values of IC 50 were 12.85,13.3 and 18.12 µg/ml. Whereas in ZnO QDs the three cell lines were arranged as DU-145, HSF and T-47D, since the values of IC50 were 17.05,32.16 and 42.59 µg/ml. Finally, it could be stated that both ZnO NP_s_ and ZnO QD_s_ showed a dose dependent response in all tested cell lines. The inhibitory percentage confirmed the results and ZnO NP_s_ showed an excellent anticancer activity against all tested cell lines compared with chemically synthesized ZnO QD_s_. These results run parallel with^36^ who stated that ZnO nanostructures can induce cytotoxic and genotoxic effects to normal mammalian cells and cancerous cells respectively. Moreover^37^, Showed that the toxic effects of ZnO in normal cells depends greatly on their size, shape, and concentration, as well as culture time and cell type. In general, ZnO exhibits concentration, size, and shape-dependent toxicity towards different cell lines. Moreover, ZnO nanorods also induced DNA damage through the ROS generation^38^. demonstrated that the dose-dependent cytotoxicity was caused by the accumulation of intracellular Zn^2+^ ions. Finally^39^, pinpointed that from (MTT) assay, cytotoxicity induced by ZnO NP_s_ was size- and dose-dependent, especially for the cells treated with smallest ZnO NP_s_ (20 nm). Moreover^40^, postulated that NPs have the ability to significantly boost the production of reactive oxygen species (ROS), which is a key component of their cytotoxic behaviors. ROS includes the superoxide radical (• O^2^), hydrogen peroxide (H_2_O_2_), and the hydroxyl radical (• OH), causing damage to cellular components such as lipids, DNA, and proteins and eventually leading to cell death^41^. shown that NPs may enter cancer cells, disperse there, and exhibit anticancer effects without the need of polymer carriers and exerting diffusion in cancer cells using fatty acids and surfactant stabilizers. However^42^, proved that ROS level increased in a time and dose-dependent manner. Consequently, it was proposed that decreased cell viability was related to oxidative stress. The cells, organelles, and enzymes would become disorganized as a result of the free radicals^43^. Mitochondria are particularly susceptible to cellular stress. Because of their high metabolic activity^44,45^.This was in accordance^21^ reports that ZnO NP_s_ lead to mitochondrial membrane depolarization in HaCaT cells and human bone marrow-derived MSCs). Both mitochondrial membrane depolarization and free radicals could trigger apoptosis via the intrinsic pathway^52^. has evaluated the cytotoxicity effects of ZnO-NP_s_ on the WEHI 164 murine fibrosarcoma cell, and in accord with the cytotoxicity assay, 250 *µ*g ml^−1^ of ZnO-NP_s_ has shown the highest toxicity on these particular cells. The calculated IC_50_ of ZnO-NP_s_ has been observed to be 212.5 *µ*g ml^−1^ compared to Our results in this research, our results are very good.

**Table 4:** Samples information of cytotoxicity test of both ZnO NP_s_ fabricated by *Padina Pavonica* extract and ZnO QD_s_ chemically synthesized against (T-47D) breast ductal carcinoma.

Compounds	Cell line	Test done	IC_50_	Unit
ZnO NP_s_	T-47 Breast ductal carcinoma	MTT assay through determination of 10 concentrations	**18.12**	µg/ml
ZnO QD_s_			**42.59**	µg/ml

**Table 5:** Cytotoxicity test of different concentrations of chemically synthesized of ZnO QD_s_ against breast ductal carcinoma (T-47D).

Concentrations	Viability %	Inhibitory %	S. D±
Control	100	0.00	0.00
0.03	98	1.86	2.04
0.1	98	1.65	1.66
0.3	98	1.48	2.46
1	97	2.27	2.19
3	90	9.50	1.38
10	91	8.78	1.96
25	85	14.63	1.82
50	63	36.60	3.72
100	37	62.63	3.84

**Table 6:** Cytotoxicity test of different concentrations of ZnO NP_s_ fabricated by *Padina Pavonica* extract against breast ductal carcinoma (T-47D).

Concentrations	Viability %	Inhibitory %	S. D±
Control	100	0.00	0.00
0.03	99.33	0.67	1.53
0.1	98.63	1.37	1.56
0.3	96.88	3.12	2.92
1	93.26	6.74	2.04
3	93.53	6.47	1.44
10	89.06	10.94	1.75
25	43.79	56.21	0.51
50	29.32	70.68	0.62
100	27.32	72.68	0.41

**Table 7:** Samples information of cytotoxicity test of both ZnO NP_s_ fabricated by *Padina Pavonica* extract and ZnO QD_s_ chemically synthesized against (DU-145) prostate cancer cell.

Compounds	Cell line	Test done	IC_50_	Unit
ZnO NP_s_	DU-145 prostate cancer cell	MTT assay through determination of 10 concentrations	13.3	µg/ml
ZnO QD_s_			17.05	µg/ml

**Table 8:** Cytotoxicity test of different concentrations of ZnO QD_s_ chemically synthesized against (DU-145) prostate cancer cell.

Concentrations	Viability %	Inhibitory %	S. D±
Control	100	0.00	0.00
0.03	100	0.14	0.13
0.1	100	0.12	1.28
0.3	95	4.11	2.03
1	95	4.09	1.73
3	92	7.52	1.88
10	91	8.09	2.05
25	35	64.87	0.67
50	23	76.37	0.28
100	20	79.72	0.56

**Table 9:** Cytotoxicity test of different concentrations of ZnO NP_s_ fabricated by *Padina Pavonica* extract against (DU-145) prostate cancer cell.

Concentrations	Viability %	Inhibitory %	S. D±
Control	100	0.00	0.00
0.03	100	0.00	1.64
0.1	99.18	0.82	2.43
0.3	98.69	1.31	2.06
1	98.57	1.43	2.04
3	97.46	2.54	1.28
10	87.20	12.8	1.74
25	19.90	80.1	0.23
50	19.44	80.56	0.46
100	16.72	83.28	0.47

**Table 10:** Samples information of cytotoxicity test of both ZnO NP_s_ fabricated by *Padina Pavonica* extract and ZnO QD_s_ chemically synthesized against HSF: human skin fibroblast.

Compounds	Cell line	Test done	IC_50_	Unit
ZnO NP_s_	HSF: Human skin fibroblast	MTT assay through determination of 10 concentrations	12.85	µg/ml
ZnO QD_s_			32.16	µg/ml

**Table 11:** Cytotoxicity test of different concentrations of ZnO QD_s_ chemically synthesized against HSF: human skin fibroblast.

Concentrations	Viability %	Inhibitory %	S. D±
Control	100	0.00	0.00
0.03	99	0.88	1.19
0.1	97	2.92	1.93
0.3	96	3.4	1.27
1	96	3.64	1.80
3	94	5.65	1.56
10	88	11.21	2.09
25	51	48.49	0.67
50	35	64.28	0.87
100	23	76.4	0.53

**Table 12:** Cytotoxicity test of different concentrations of ZnO NP_s_ fabricated by *Padina Pavonica* against HSF: human skin fibroblast.

Concentrations	Viability %	Inhibitory %	S. D±
Control	100	0.00	0.00
0.03	97.55	2.45	2.72
0.1	96.12	3.88	1.91
0.3	93.66	6.34	2.96
1	92.37	7.63	2.02
3	87.69	12.31	2.01
10	77.34	22.66	1.65
25	31.62	68.38	0.33
50	27.29	72.71	0.53
100	25.14	74.86	0.63

## Conclusion

The biogenic approach is not only environmentally friendly and nontoxic, but also remarkably simple as compared to other approaches. Zinc Oxide quantum dots have been prepared by a chemical precipitation technique and we successfully biosynthesized ZnO NPs by a green method. From photocatalytic activity and kinetics rates of pure MB, Rh 6G and Fluorescein dyes and also immersed in ZnO QD_s_ and ZnO NP_s_, we found that the photodegradation of dyes as a pollutant in the presence of ZnO nanostructures under UV-irradiation is faster than photodegradation of pure dyes which indicate that the ZnO NP_s_ and ZnO QD_s_ are the good removal of pollutant dyes. Also, MB dye has a higher photodegradation percentage in the presence of ZnO QD_s_ as a catalyst than Rh 6G and Fluorescein dyes in the presence of ZnO NP_s_. So, ZnO QD_s_ is the best catalyst of a pollutant removal than ZnO NP_s_ due to the smaller size of QD_s_ facilities the separation of photogenerated electron-hole pairs, which are crucial for the degradation process. The optical band gap energy of ZnO NP_s_ and ZnO QD_s_ is decrease by increasing irradiation time in presence dyes. The above prepared nanomaterials were applied to be anticancer, where the ZnO NP_s_ behave as a good anticancer nanomaterial than ZnO QD_s_. This means ZnO NP_s_ are superior for anticancer applications if compared with ZnO QDs.

## Data Availability

The supporting data of the current study are available from the corresponding author on reasonable request.
